# Lyl1-deficiency promotes inflammatory responses and increases mycobacterial burden in response to *Mycobacterium tuberculosis* infection in mice

**DOI:** 10.3389/fimmu.2022.948047

**Published:** 2022-09-02

**Authors:** Shelby-Sara Jones, Mumin Ozturk, Nathan Scott Kieswetter, Sibongiseni K. L. Poswayo, Rudranil Hazra, Ousman Tamgue, Suraj P. Parihar, Harukazu Suzuki, Frank Brombacher, Reto Guler

**Affiliations:** ^1^ International Centre for Genetic Engineering and Biotechnology, Cape Town Component, Cape Town, South Africa; ^2^ Department of Pathology, University of Cape Town, Institute of Infectious Diseases and Molecular Medicine (IDM), Division of Immunology and South African Medical Research Council (SAMRC) Immunology of Infectious Diseases, Faculty of Health Sciences, University of Cape Town, Cape Town, South Africa; ^3^ Wellcome Centre for Infectious Diseases Research in Africa (CIDRI-Africa), Institute of Infectious Disease and Molecular Medicine (IDM), Faculty of Health Sciences, University of Cape Town, Cape Town, South Africa; ^4^ Epigenomics & Single Cell Biophysics Group, Department of Cell Biology Faculty of Science, Radboud University, Nijmegen, Netherlands; ^5^ Department of Biochemistry, Faculty of Sciences, University of Douala, Douala, Cameroon; ^6^ Laboratory for. Cellular Function Conversion Technology RIKEN Center for Integrative Medical Sciences, Yokohama, Japan

**Keywords:** *Mycobacterium tuberculosis*, lymphoblastic leukemia 1, neutrophilic inflammation, transcription factor, innate immunity

## Abstract

Lymphoblastic leukemia 1 (Lyl1) is a well-studied transcription factor known to exhibit oncogenic potential in various forms of leukemia with pivotal roles in hematopoietic stem cell biology. While its role in early hematopoiesis is well established, its function in mature innate cells is less explored. Here, we identified Lyl1 as a drastically perturbed gene in the *Mycobacterium tuberculosis* (*Mtb*) infected mouse macrophage transcriptome. We report that Lyl1 downregulation upon immune stimulation is a host-driven process regulated by NFκB and MAP kinase pathways. Interestingly, Lyl1-deficient macrophages have decreased bacterial killing potential with reduced nitric oxide (NO) levels while expressing increased levels of pro-inflammatory interleukin-1 and CXCL1. Lyl1-deficient mice show reduced survival to *Mtb* HN878 infection with increased bacterial burden and exacerbated inflammatory responses in chronic stages. We observed that increased susceptibility to infection was accompanied by increased neutrophil recruitment and IL-1, CXCL1, and CXCL5 levels in the lung homogenates. Collectively, these results suggest that Lyl1 controls *Mtb* growth, reduces neutrophilic inflammation and reveals an underappreciated role for Lyl1 in innate immune responses.

## Introduction

Lymphoblastic leukemia 1 (Lyl1) is a Class II basic helix-loop-helix (bHLH) transcription factor that is highly expressed in both T-cell Acute Lymphoblastic Leukemia (T-ALL) (1) and Acute Myeloblastic Leukemia (AML) (2). It was originally discovered upon ectopic expression in human t(7;19)(q35;p13)-positive T-ALL (3) and later described to serve a significant role during late hematopoiesis, including B- and T-cell reconstitution (4). Zohren et al. further demonstrated that Lyl1 controls the maintenance of uncommitted T-cell progenitors during T-cell development in the thymus (5). Thus, Lyl1 was originally characterized as a T-cell-associated oncogene (3). However, a subsequent study by Lukov et al. has shown that Lyl1 is not directly oncogenic. Rather, an overexpression of Lyl1 increases proliferation, suppresses apoptosis of progenitor cells, and thus, predisposes mice towards lymphoma after which secondary mutations occur for cancer development (6). Aside from lymphoid lineage-specific roles, recent studies show that Lyl1 modulates the development of primitive macrophage progenitors, and microglia at early embryonic stages (7) and compensates for the loss of stem cell leukemia (SCL) in megakaryopoiesis and platelet function (8). In contrast to all the discovered important functions of Lyl1 in hematopoietic development, Lyl1-deficient mice are viable with a milder phenotype on the immune system in adult mice (4, 5). Stem cell leukemia (SCL) is a bHLH transcription factor with high amino acid sequence similarity to Lyl1 and an essential regulator of the hematopoiesis (4, 9). Previous studies have suggested that both transcription factors can compensate for each other; however, Lyl1 cannot rescue the early lethality of SCL deletion in the embryonic stage (10, 11). Considering the minor effects of Lyl1 deletion in adult hematopoiesis, this study aims to evaluate the outcomes of Lyl1 deficiency in the presence of bacterial infections.

*Mycobacterium tuberculosis* (*Mtb*) remains one of the leading fatal disease-causing microbes to date. Its remarkable ability to manipulate and exploit cellular host responses for its survival and virulence continues to encourage further investigation into novel therapeutics and treatment strategies for TB (12–18). Since Robert Koch’s tubercle bacillus discovery in 1882 (19), extensive research has been dedicated to the treatment of tuberculosis (TB). However, as we move further into the twenty-first century with various TB treatment regimens in the clinic, the tubercle bacillus is continuously developing resistance, intensifying its virulence (20). An emerging field of host-directed therapy (HDT) has been widely studied as a means of circumventing the bacteria’s manipulative and adaptive nature (12–18). These studies aim to develop adjunctive anti-TB therapies that would enhance the host immune response to effectively eradicate the bacteria while reducing pulmonary pathology (12, 18, 21). Alveolar macrophages are the primary cells that phagocytose infectious *Mtb* and the infection cycle of *Mtb* is greatly decided in the macrophages (22). The use of genome-wide gene expression analysis during infection of macrophages with *Mtb* would aid in identifying complex host-pathogen interplay and potential HDT targets for TB. The FANTOM (Functional Annotation of Mammalian Genome) consortium has implemented single-molecule Cap Analysis Gene Expression (CAGE) to provide a transcriptional network of various cell states in both human and murine cells (23–26). Interestingly, an in-depth analysis of the FANTOM5 dataset revealed the downregulation of Lyl1 gene expression in *Mtb*-infected IFNγ and IL-13/IL-14-stimulated bone marrow-derived macrophages.

In this study, we describe underexplored functional aspects of Lyl1 during *Mtb* infection using *in vitro* and *in vivo* mouse models for TB. We describe the host-regulation of Lyl1 expression by MAPk and NFκB signaling pathways. Furthermore, we show significant host susceptibility to *Mtb* HN878 in the absence of Lyl1 and suggest increased neutrophilic inflammation and pro-inflammatory cytokine/chemokine expression as key drivers of susceptibility. Therefore, given the significant role of Lyl1 during *Mtb* infection, our data highlight the implication of Lyl1-associated pathways in TB protection.

## Materials and methods

### Mice

The Lyl1*^-/-^* mouse, generated by the Margaret A. Goodell laboratory at Baylor College of Medicine, Houston, Texas (27), back-crossed to the C57BL/6 strain for at least ten generations. The strain was kindly provided by Barbara L Kee, University of Chicago, Illinois. Once received at the University of Cape Town (UCT), Lyl1*^-/-^* was back-crossed with in-house C57BL/6J strains for three generations to generate Lyl1^-/-^, Lyl1^-/+^ and littermate control, Lyl1^+/+^ in the Animal Research Facility, UCT. Animals were housed, monitored, and experimentally handled within strict accordance with the guidelines approved by the Animal Research Ethics Board of UCT. All experiments included mice aged 8-12 weeks and were sex matched.

### Ethical statement

All animals used in this study were subjected to experimental procedures that were in strict accordance with the South African National Standard (SANS 10386:2008) and the Animal Research Ethics Committee of the Faculty of Health Sciences, University of Cape Town (Protocol Permit No: 015/040 and 019/023). The recruitment of healthy volunteers for this study was approved by the Human Ethics Committee Faculty of Health Sciences, University of Cape Town, Cape Town (HREC Ref Number: 140 732/2015).

### BMDM-MDM generation, *in vitro* infection, and pathway activation/inhibition

Bone marrow-derived macrophages (BMDM) were generated from the femurs of 8-12 week old mice as previously described (28). Post differentiation, a total of 7.5x10^5^ were seeded in 24-well plates (Nunc, Roskilde, Denmark) for downstream infection or pathway activation/inhibition. Overnight adhered cells were either infected with BCG, H37Rv, HN878, CDC1551, N72, heated-killed *Mtb* at a multiplicity of infection (MOI) 1, or *Lm* at MOI:10. Uptake of bacteria was determined by examining bacterial loads 4 hours post infection. Lysed BMDM (in 0.05% Triton X-100) were subjected to 10-fold dilutions and plated on Middlebrook 7H11 agar plates supplemented with 10% OADC and 0.5% glycerol. Plates were incubated at 37°C for 14-21 days and the colony-forming units (CFU) enumerated. Cells were collected at indicated time points for RNA extraction. Supernatants were collected for protein analysis by ELISA.

Monocyte-derived macrophages (MDM) were generated from Leukopaks obtained from Western Province Blood Service. Briefly, Leukopak was diluted 1:1 with phosphate-buffered saline (PBS) containing 2% fetal bovine serum (Gibco, ThermoFisher, Massachusetts) and centrifuged at 500g for 25 minutes with brakes off in Leucosep tubes (Greiner Bio-one, Frickenhausen, Germany) with Histopaque 1077 (Sigma Aldrich, St. Louis, Missouri). The buffy coat is removed by Pasteur pipette and washed twice with PBS+2%FBS at 120g to remove platelets. Peripheral blood mononuclear cells were counted and subjected to pan monocyte isolation kit (Miltenyi Biotec, Gladbach, Germany) according to the manufacturer’s instructions. Isolated monocytes were seeded in 60 mm Nunc cell culture dishes (ThermoFisher, Massachusetts) at a concentration of 1x10^6^ cells/ml in RPMI 1640 media (ThermoFisher, Massachusetts) supplemented with 10% human AB serum (Sigma Aldrich, St. Louis, Missouri), 50 U/ml penicillin G (ThermoFisher, Massachusetts), 50 µg/ml streptomycin (ThermoFisher, Massachusetts) and 50 ng/ml recombinant human M-CSF (Peprotech, Rocky Hill, New Jersey) for 7 days. MDM were harvested after 20 minutes of incubation in Accutase^®^ (Sigma Aldrich, St. Louis, Missouri) solution. MDM were seeded in tissue culture-treated 96-well flat-bottom plates (Costar^®^, Corning) at a concentration of 1x10^6^ cells/ml without the antibiotics for downstream infection experiments.

For pathway activation, BMDM were seeded in 24- (7.5x10^5^) and 6-well (3x10^6^) plates for RNA and protein collection, respectively. BMDM were subjected to cyclic Guanosine Monophosphate-Adenosine Monophosphate, cGAMP (Sigma Aldrich, St. Louis, Missouri), CpG oligodeoxynucleotide, CpG ODN 1668 (*Invivo*Gen, San Diego, California), Lipopolysaccharide, LPS (Sigma Aldrich, St. Louis, Missouri), Muramyl Dipeptide, MDP (Sigma Aldrich, St. Louis, Missouri), Pam3CysSerLys4, Pam_3_CSK_4_ (Tocris, Bristol, UK), Trehalose-6,6-dimycolate, TDM (Sigma Aldrich, St. Louis, Missouri) at indicated titrated concentrations.

For pathway inhibition, BMDM were exposed to 7.5 µM Bay11-7082 (NFκB inhibitor; Sigma Aldrich, St. Louis, Missouri), 5 µM SB 203580 (p38 MAPk inhibitor; Tocris, Bristol, UK), 10 µM SP600125 (JNK MAPk inhibitor; Sigma Aldrich, St. Louis, Missouri), and/or 10 µM FR180204 (ERK1/2 MAPk inhibitor; Sigma Aldrich, St. Louis, Missouri) as well as 10 µM SB 747651A dihydrochloride (MSK1/2 inhibitor; Tocris, Bristol, UK) for one hour prior to 100 ng/ml LPS stimulation. Cells for RNA extraction were collected at indicated time points. Nascent RNA was captured using the Click-iT™ Nascent RNA capture kit (Invitrogen, Waltham, Massachusetts).

### Western blot

Western blot analysis was performed as previously described (29). Cell lysate protein content was determined using the BCA Protein Assay Kit (Thermo Fisher Scientific, Waltham, Massachusetts) after which a total of 20-40 µg of protein was used to determine expression patterns. The membrane was probed with either p38 MAPK (D13E1) XP^®^, Phospho-p38 MAPK (Thr180/Tyr182) (D3F9) XP^®^, SAPK/JNK Antibody, Phospho-SAPK/JNK (Thr183/Tyr185) (G9), NFκB p65 (D14E12) XP^®^, Phospho-NFκB p65 (Ser536) (93H1) XP^®^ (all from Cell Signaling Technology, Danvers, Massachusetts) NFκB p50/p105 (Clone 1N19, ZooMAb^®^) (Sigma Aldrich, St. Louis, Missouri) or GAPDH (Sant Cruz Biotechnology, Dallas, Texas) primary antibodies and captured using either goat anti-rabbit IgG H&L (HRP) pre-absorbed or goat anti-mouse IgG H&L (HRP) pre-absorbed (both from Abcam, Cambridge, UK). Immunoblots were developed using the KPL LumiGLO^®^ Reserve Chemiluminescent Substrate Kit (SeraCare Life Sciences, Milford, Massachusetts) on the iBright FL1000 Imaging System (Thermo Fisher Scientific, Waltham, Massachusetts). Densitometry analysis was performed using the built-in iBright FL1000 Imaging System after which each band was normalized to GAPDH.

### *Mtb* infection in mice (*in vivo*)

Anesthetized mice were intranasally infected with *Mtb* HN878 in sterile saline by administering 25 µl per nasal cavity. The actual inoculum dose was 100 – 200 CFU/mouse as determined by lung bacillary uptake at 24 hrs post-infection in 3-4 mice. Bacterial loads, physiological parameters, flow cytometric, and histopathological analyses were performed on indicated organs as previously directed (30).

### Bacterial loads in BMDM and homogenates

At various indicated timepoints, lysed BMDM (in 0.05% Triton X-100) and organ homogenates were subjected to 10-fold dilutions and plated on Middlebrook 7H11 agar plates supplemented with 10% OADC and 0.5% glycerol. Plates were incubated at 37°C for 14-21 days and the colony-forming units (CFU) for cell culture and organ enumerated.

### Quantitative real-time polymerase chain reaction (qRT-PCR)

Plated cells were collected with 350 µl of RLT lysis buffer designed to extract RNA using the RNeasy Mini Kit (Qiagen, Hilden, Germany). RNA was extracted according to the manufacturer’s instructions and normalized to 300 ng for downstream cDNA synthesis. RNA was reverse transcribed using the Transcriptor First Strand complementary DNA (cDNA) Synthesis Kit (Roche, Basel, Switzerland) using both anchored oligo dT primers and random hexamer primers according to the manufacturer instructions. Various transcripts, listed in **Supplementary Table 1**, were amplified by qPCR using LightCycler^®^ 480 SYBR Green I Master Mix (Roche, Basel, Switzerland), in the LightCycler^®^ 480 Instrument II (Roche, Basel, Switzerland). All targeted expressions were normalized to Hprt expression levels.

### Measurement of nitric oxide and cytokine/chemokine in culture supernatants and homogenates

Nitrite levels in infected cell culture supernatants were measured using the Griess reagent assay. Briefly, cell supernatants were incubated with 1% sulfanilamide in 2.5% phosphoric acid for 10 minutes at room temperature in the dark, followed by 0.1% naphthyl-ethylene-diamine in 2.5% phosphoric acid for another 10 minutes. Cytokines and chemokines from BMDM cell culture and organ homogenates were examined using the standard sandwich enzyme-linked immunosorbent assay (ELISA) protocol. Capture and biotin antibodies were obtained from either BD Biosciences (Franklin Lakes, New Jersey), BioLegend (San Diego, California), or R&D Systems (Minneapolis, Minnesota) using either KPL TMB Microwell Peroxidase Substrate (SeraCare Life Sciences, Milford, Massachusetts) for streptavidin-HRP conjugates or 1 mg/ml p-nitrophenyl phosphate disodium salt hexahydrate (Sigma Aldrich, St. Louis, Missouri) for streptavidin-AP conjugates. Optical density was measured using the VersaMax™ microplate spectrophotometer (Molecular Devices, San Jose, California).

### Flow cytometry

Collected organs were subjected to collagenase digestion in preparation for single-cell suspension, followed by mechanically passing through a 100 µm and 70 µm strainer sequentially as previously described (30). Erythrocytes were lysed using red blood cell (RBC) lysis buffer (155 mM NH4Cl, 12 mM NaHCO3, 0·1 mM EDTA). A single-cell suspension (1x10^6^ cells) from indicated organs were stained for the following surface markers suspended in PBS supplemented with 1% BSA and 0.1% NaN_3_, purchased from either BD Biosciences (Franklin Lakes, New Jersey), BioLegend (San Diego, California) or eBioScience (San Diego, California): PD-1 (Clone 29F.1A12 FITC, Biolegend); CD4 (Clone RM4-5 BV510, BD Biosciences); CD44 (Clone IM7 PE, BD Biosciences); NK1.1 (Clone PK136 APC-Cy7, BD Biosciences); CD3 (Clone 500A2 AF700, BD Biosciences); CXCR5 (Clone 2G8 PE-Cy7, BD Biosciences); CD62L (Clone MEL-14 V450, BD Biosciences); CD19 (Clone 1D3 PerCPCy5.5, BD Biosciences); CD8 (Clone 53-6.7 APC, BD Biosciences); KLRG1 (Clone 2F1/KLRG1 BV785, Biolegend); CD64 (Clone X54-5/7 PeCy7, BioLegend); Ly6C (Clone AL-21 PerCPCy5.5, BD Biosciences); CD11b (Clone M1/70 V450, BD Biosciences); MHC II (Clone M5/114.15.2 AF700, BioLegend); CD11c (Clone HL3 APC, BD Biosciences); SiglecF (Clone E5-2440 APC-Cy7, BD Biosciences); Ly6G (Clone 1A8 FITC, BD Biosciences); MerTK (Clone 108928 BV786, BD Biosciences); CD103 (Clone M290 PE, BD Biosciences); F4/80 (Clone BM8 PeCy7, eBioscience); CD169 (Clone SER-4 APC-eFluor780, eBioscience). Cells were stained and acquired according to (30) using the BD LSR Fortessa and the data analysis was performed with FlowJo v10.6.1 Software (Treestar, Ashland, Oregon) following the gating strategies outlined in **Supplementary Figures 5**–**10**.

### Histopathology and immunohistochemistry

Upon animal euthanasia and organ excision, a fraction of the indicated organ was submerged in formalin solution (10% formaldehyde in 1X PBS) for tissue fixation after which 3 µm thick sections were stained with hematoxylin and eosin (H&E) for histopathological analyses. Lung tissue was also processed for immunohistochemistry using using iNOS antibody (ab3523, Abcam, Cambridge, UK). Stained sections were mounted onto microscopic slides using a xylene-based mounting medium for image acquisition and quantification using the Nikon Eclipse 90i microscope with the Nikon NIS-Elements advanced imaging software (Nikon Corporation, Tokyo, Japan). Lung alveolar spacing was quantified by subtracting the entire lung surface area from the lung tissue occupied by cells. The data represents the sections of the lung that is free from cells and thus indicative of free alveolar space. The threshold intensity for the iNOS stain was determined using the iNOS antibody control images after which the thresholding value was maintained across all lung sections for each experiment. The data represents the lung sections that has been positively stained.

### Data deposition

The FANTOM5 CAGE data is publicly available (http://fantom.gsc.riken.jp/5) and can be interactively explored using the Zenbu portal (https://fantom.gsc.riken.jp/zenbu/). Data were manually extracted from additional published databases, including the TB Gambia Cohort (PMID 22046420), TB SA and UK Cohort (PMID 20725040), and the Viral USA Cohort (PMID 26070066), organized and sorted using Microsoft Excel^®^ (Redmond, Washington) and represented using GraphPad Prism 6 (San Diego, California).

### Statistical analysis

All data represented was analyzed using GraphPad Prism 6.0 (San Diego, California) with the use of the student *t-test* (two-tailed with equal variance). A *p value of less than 0.05 was considered significant, depicting **p < 0.01, ***p < 0.001 and ****p < 0.0001.

## Results

### Lyl1 expression is significantly downregulated in response to bacterial infection and LPS stimulation

**Figure 1:**
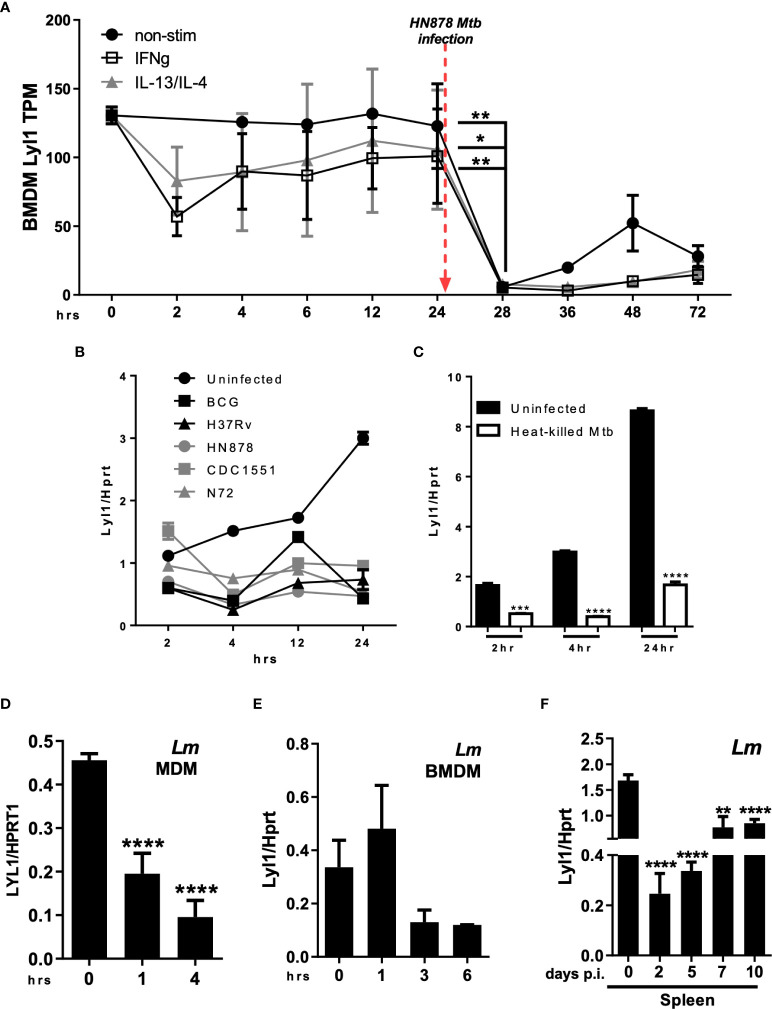
Regulation of Lyl1 expression is a host-driven mechanism. **(A)** Expression kinetics (represented as Tags Per Million (TPM)) of Lyl1 in *Mtb* HN878 infected mouse bone marrow-derived macrophage (BMDM) data were extracted from the FANTOM5 mouse macrophages dataset. **(B)** Lyl1 mRNA expression was measured by RT-qPCR, relative to the Hprt housekeeping gene, in BMDM infected with various *Mtb* strains, as well as **(C)** heat-killed *Mtb* at MOI:1. **(D)** MDM derived from healthy participants were infected with *Lm* with MOI:10 to measure LYL1 mRNA expression kinetics. **(E)** BMDM were infected with *Listeria monocytogenes* (*Lm*) with MOI:10 for RNA collection. **(F)** A total of 2x10^5^ CFU/mouse of *Lm* was intraperitoneally injected into C57BL/6 mice (*n* = 3 mice/group) after which the spleen and liver were collected at indicated time points for RNA isolation. Error bar denotes Mean ± SEM. Data shown are representative of 2-4 independent experiments. Unpaired student t-test analysis at *p < 0.05, **p < 0.01, ***p < 0.001, ****p < 0.0001 to determine significance.

Data extracted from FANTOM5, demonstrating CAGE sequencing of diverse cellular states, showed a significant reduction in Lyl1 expression in all macrophage subsets (unstimulated, M1 (IFNγ stimulated) and M2 (IL-13/IL-4 stimulated) following *Mtb* HN878 infection *in vitro* (**Figure 1A**) (23, 25); while the downregulation was not observed in uninfected macrophages (**Supplementary Figure 1A**). HN878 infection revealed stable cell viability over time (**Supplementary Figure 1B**). A similar expression pattern was also observed in human monocyte-derived macrophages (MDM) infected with *Mtb* HN878 (**Supplementary Figure 1C**). It is evident that Lyl1 downregulation is independent of *Mtb* virulence given the decreased expression of Lyl1 mRNA across various *Mtb* strains as well as BCG (**Figure 1B**) and heat killed *Mtb* (**Figure 1C**). Additionally, the FANTOM5 data revealed the downregulation of Lyl1 in human MDM following LPS stimulation (**Supplementary Figure 1D**). Lyl1 downregulation was also observed in various organs of LPS-treated mice (**Supplementary Figure 1E**). Moreover, the downregulation of Lyl1 was observed in *Listeria monocytogenes* (*Lm*) infected human MDM (**Figure 1D**). In parallel, we noted a similar pattern of downregulation in *Lm* infected*-*mouse macrophages, albeit not statistically significant (**Figure 1E**). In a murine *Lm in vivo* model, a downregulation pattern was steadily observed (**Figure 1F**), although this downregulation was not observed in later timepoints during the slow progressing *Mtb* infection *in vivo* (**Supplementary Figure 1F**). Collectively, this data suggests that the downregulation of Lyl1 may not be a consequence of an immune evasion mechanism caused by *Mtb*, but rather a host regulatory mechanism to potentially control bacterial infection. This is evident in the reduction of Lyl1 mRNA levels during *Mtb* and *Lm* infection as well as during LPS-treatment in both *in vitro* and *in vivo* murine models. Additionally, this observation was noted *in vitro* in human MDM.

We further explored Lyl1 expression in the whole blood transcriptome of various cohorts. Lyl1 levels remained unchanged in different cohorts of active TB, latent TB, and healthy individuals, yet influenza A and rhinovirus infections resulted in a transient decrease in Lyl1 expression (**Supplementary Figure 2**). Taken together, the Lyl1 downregulation pattern implies an early host response that activates the downstream immune response cascade.

### MAPk and NFκB signaling pathways are key regulators of Lyl1 expression, yet they are not regulated by Lyl1

**Figure 2:**
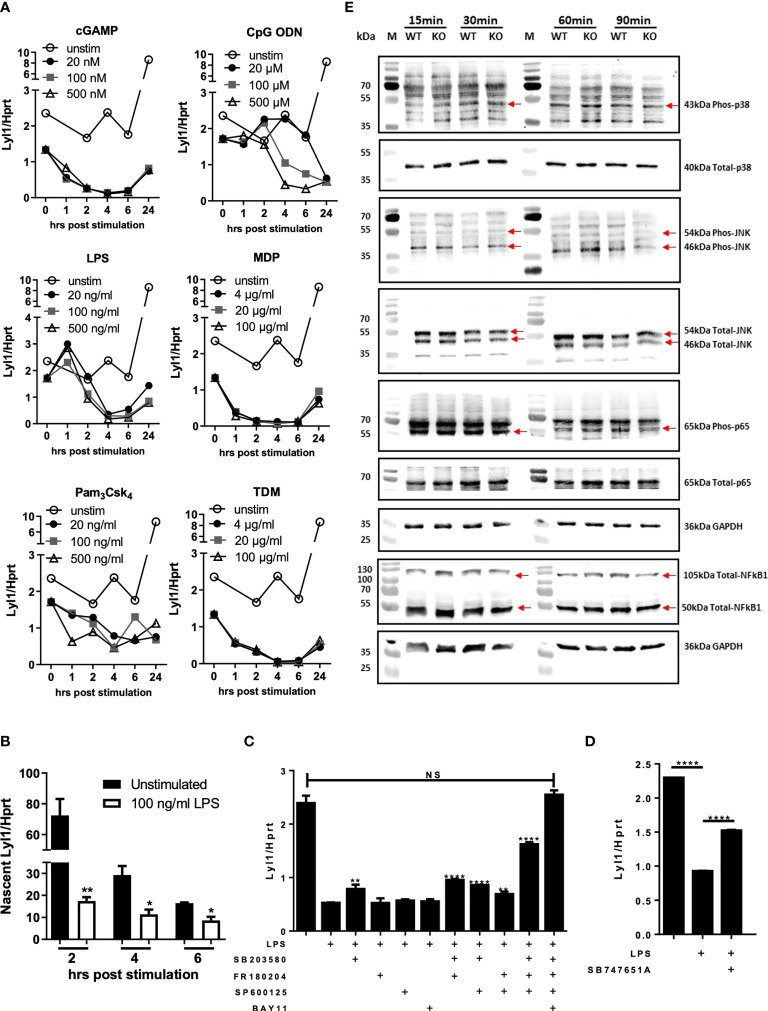
Lyl1 expression is downregulated by MAPk and NFkB signaling pathways in macrophages. **(A)** Various intracellular signaling pathways were activated in BMDM cells using cGAMP, CpG ODN, LPS, MDP, Pam3Csk4, or TDM at indicated concentrations. RNA was collected at 0, 1, 2, 4, 6, and 24 hrs hours and Lyl1 mRNA expression was investigated by RT-qPCR, relative to the Hprt housekeeping gene. **(B)** BMDM were stimulated with LPS (or unstimulated) and simultaneously tagged with biotinylated ethynyl uridine (EU) followed by RNA pulldown for nascent RNA collection. RT-qPCR was performed on synthesized cDNA to investigate Lyl1 mRNA expression. **(C)** BMDM were exposed to various inhibitor cocktails including 5 µM SB203580 (p38 MAPk inhibitor), 10 µM FR180204 (ERK1/2 MAPk inhibitor), 10 µM SP600125 (JNK MAPk inhibitor), and 7.5 µM BAY11 7084 (NFkB inhibitor) as well as **(D)** 10 µM SB747651A (MSK1/2 inhibitor) for 1 hour prior to 100 ng/ml LPS stimulation for 4 hrs. Cells were collected for RNA isolation and cDNA synthesis after which Lyl1 expression was investigated by RT-qPCR. Asterisks over the bars indicate statistical significance upon comparisons of inhibitor-treated samples *versus* LPS stimulated sample. **(E)** WT and Lyl1^-/-^ BMDM were stimulated with 100 ng/ml LPS, and protein lysates were collected at indicated time points after which western blot analysis on 30µg loaded protein was performed using phospho-p38 MAPk, total-p38 MAPk, phospho-SAPK/JNK MAPk, total-SAPK/JNK MAPk, phospho-RelA (p65), total-RelA (p65), total-NFκB1 (p50/p105), as well as GAPDH primary antibodies. Data shown are representative of two independent experiments. Unpaired student t-test analysis at *p < 0.05, **p < 0.01, ****p < 0.0001 to determine significance, ns, not significant.

Given the potential host regulative mechanism linked to Lyl1 expression, we next aimed to investigate various intracellular signaling cascades regulating Lyl1 expression in response to agonist stimulation of pattern recognition receptors that are mainly activated by bacterial infections (31). Using a dose-response assessment, Lyl1 expression in BMDM across various activated signaling pathways was examined. These included the STING (cGAMP stimulation), TLR9 (CpG ODN stimulation), TLR4 (LPS stimulation), NOD2 (MDP stimulation), TLR2 (Pam_3_Csk_4_ stimulation), and the Mincle pathways (TDM stimulation) of which Lyl1 downregulation was observed in all pathways (**Figure 2A**). It is evident that the regulation of Lyl1 mRNA is not pathway-specific, but rather regulated by potential master transcription factors or post-transcriptional mechanisms. Therefore, we initially investigated whether the regulation of Lyl1 mRNA occurs pre- or post-transcriptionally by examining nascent RNA. The downregulation of nascent RNA upon LPS stimulation confirmed pre-transcriptional regulation of Lyl1 downregulation (**Figure 2B**) after which various MAPk and NFκB inhibitor combinations were used to determine the potential regulatory component of Lyl1 expression (**Figures 2C, D**). Here, we demonstrate a co-regulatory function of NFκB and MAPk signaling on Lyl1 expression, collectively (**Figure 2C**). With inhibition of all MAPk subunits and NFκB prior to LPS stimulation, Lyl1 expression was comparable to that of unstimulated. This was further supported through MSK1/2 inhibition, a MAPk signaling downstream kinase, proving partial recovery of Lyl1 expression (**Figure 2D**).

We later aimed to investigate the autoregulative feedback loop potential of Lyl1 by exploring MAPk and NFκB activation patterns in the absence of Ly11. Since Lyl1 expression is regulated by MAPk signaling and NFκB transcription factor in addition to published data proving the interaction between Lyl1 and MAPk (32) as well as Lyl1 and NFκB (33), we hypothesized that Lyl1 in turn regulates these master signaling pathways. However, p38 MAPk, JNK/SAPK MAPk, NFκB1 (p50/p105), and RelA (p65) (**Figure 2E**), all displayed similar phosphorylation/protein expression patterns in BMDM independent of Lyl1. Together, these data demonstrate Lyl1 regulation by host signaling pathways including MAPk and NFκB activity, while showing that Lyl1 deficiency does not affect activation of JNK, p38, p65 pathways, and p50/p105 protein levels.

### Lyl1 is required to control *Mtb* infection in macrophages *in vitro*

**Figure 3:**
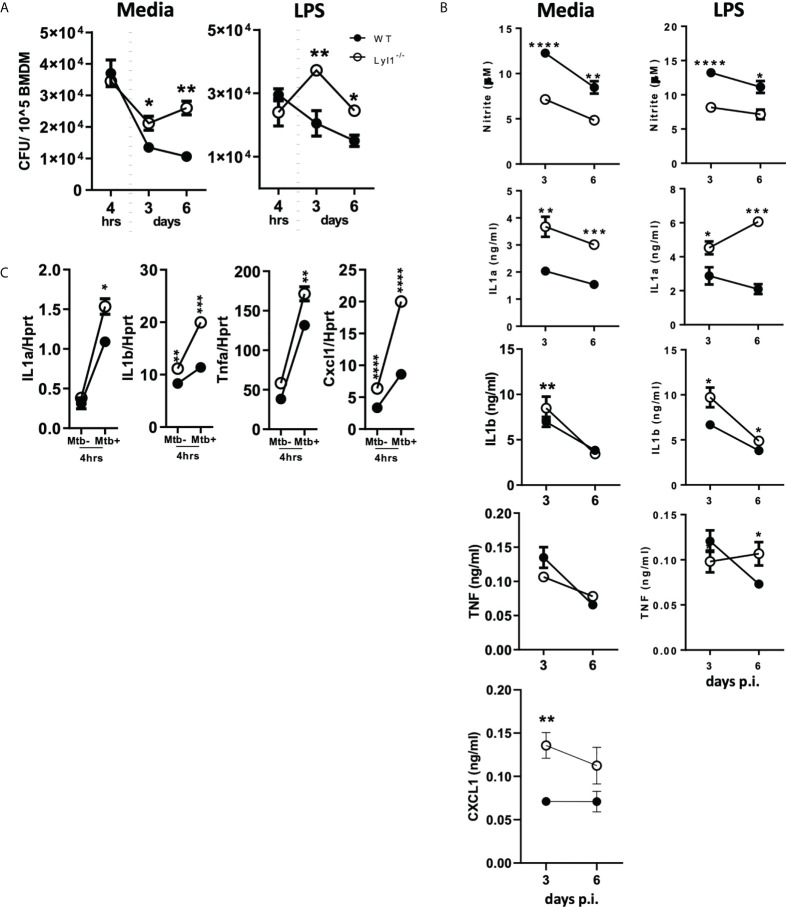
Lyl1-deficiency increases macrophage bacterial burden with differential effects on proinflammatory gene expression in response to hypervirulent Mtb HN878 *in vitro*. **(A, B)** Wild-type (WT) and Lyl1^-/-^ bone marrow-derived macrophage (BMDM) cells were exposed to either media or 100 ng/ml LPS after which they were infected with Mtb HN878 with MOI:1. **(A)** Lysed cells (3 or 6 days post-infection) were plated for intracellular bacterial burden and **(B)** supernatants (3 or 6 days post-infection) were collected for cytokine chemokine production by ELISA. Nitrite levels were measured by Griess assay. **(C)** RNA was collected from WT and Lyl1^-/-^ BMDM 4 hrs post-Mtb HN878 (MOI:1) infection, synthesized to cDNA by two-step PCR, and qPCR performed for mRNA expression. Data represented demonstrates technical replicates and the error bar denotes Mean ± SEM. Data shown are representative of 2-4 independent experiments. Unpaired student t-test analysis at *p < 0.05, **p < 0.01, ***p < 0.001, ****p < 0.0001 to determine significance.

Databases, including the Tabula Muris (34), show that Lyl1 is mainly expressed in monocyte populations in mouse lung tissue (**Supplementary Figure 1G**). Therefore, the downregulation of macrophage Lyl1 expression in response to *Mtb* HN878 (**Figure 1**) prompted further investigation into the effects of *Mtb* infection on Lyl1-deficient macrophages. Since our data suggest that Lyl1 expression is host-regulated, with no difference in mycobacterial uptake between both groups (4 hours post infection), it was interesting to observe a significantly increased bacterial burden in Lyl1-deficient BMDM when compared to wild-type BMDM in unstimulated and LPS pre-stimulated conditions at 3 and 6 days post-infection (**Figure 3A**). Notably, Lyl1-deficient BMDM showed decreased nitric oxide (NO) but increased IL-1 and CXCL1 chemokine response with no clear effects on TNF expression (**Figures 3B, C**). Therefore, we demonstrate that despite the host-driven decreased levels of Lyl1 during *Mtb* infection, complete deletion of Lyl1 leads to increased mycobacterial burden with attenuated NO response suggesting the requirement of Lyl1 to limit *Mtb* bacterial growth in macrophages.

### Lyl1-deficient mice are highly susceptible to chronic hypervirulent *Mtb* HN878 infection *in vivo*

Previous studies have shown that Lyl1 is an important factor for early hematopoiesis and lymphoid engraftment (4, 27) with minor effects on the adult immune system. Initially, we extensively scrutinized Lyl1 deficiency in adult mice. Lyl1 deficient mice did not show abnormal phenotype in terms of cellularity in various tissues or immune cell populations in the lung, spleen, mediastinal lymph node, and liver (**Supplementary Figures 3**, **4**). Given the pivotal role of Lyl1 in T-cell maturation, interestingly Lyl1 is lowly expressed in the adult thymus with the highest expression observed in the spleen (**Supplementary Figure 3A**). The lung morphology of Lyl1^-/-^ mice looked similar to their WT counterparts (**Supplementary Figures 3F, G**). As previously reported (5), we observed decreased thymocytes in the CD4-CD8 double negative (DN) stage in Lyl1^-/-^ mice; albeit with no noticeable effects on mature T cells in the thymus or other organs (**Supplementary Figure 4E**).

**Figure 4:**
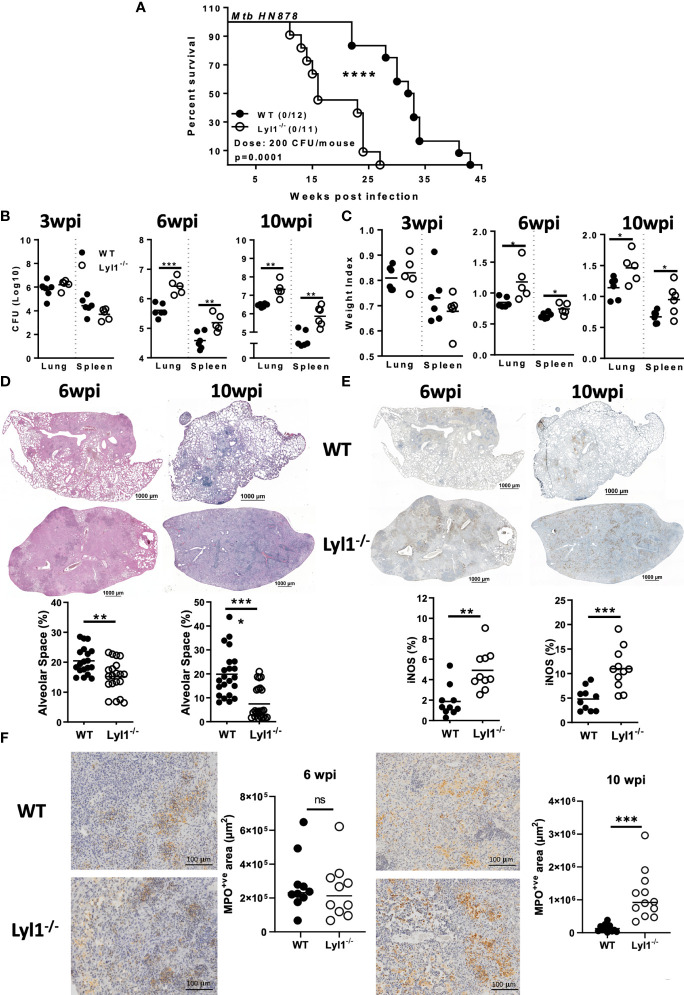
Lyl1 deletion renders mice more susceptible to Mtb HN878 infection with increasing lung and spleen bacterial burden. **(A)** Survival study by intranasally administering Mtb HN878 at 200 CFU/mouse (n = 11-12 mice/group). Mantel-Cox survival analysis is performed with log-rank test P = 0.0001. **(B-E)** Littermate control (WT) and Lyl1^-/-^ mice were infected with ~100 CFU/mouse intranasally with Mtb HN878 (n = 5-6 mice/group) and sacrificed at 3-, 6- or 10- weeks post-infection to determine **(B)** lung and spleen CFU burden as well as **(C)** lung and spleen weight index. **(D)** Representative lung histopathology sections (x20 magnification) for H&E (scale bar = 1000µm) with quantified alveolar spaces from 4 deep cut lung sections per mouse (30µm apart). Each plot represents lung sections that are free from cells (%) and thus indicative of alveolar space. **(E)** Representative iNOS immunohistochemistry lung section (x10 magnification) with quantified iNOS positive areas from two deep cut lung sections per mouse (30µm apart). **(F)** Representative myeloperoxidase (MPO) immunohistochemistry lung section (x40 magnification) with quantified MPO positive areas from two deep cut lung sections per mouse (30µm apart). Line denotes Mean. Data shown are representative of 2-3 independent experiments. Unpaired student t-test analysis at *p < 0.05, **p < 0.01, ***p < 0.001, ****p < 0.0001 to determine significance, ns, not significant.

Considering the outcome of Lyl1 deficiency at the macrophage level, we further investigated the effect of Lyl1 deficiency at the tissue/organism level during *Mtb* HN878 infection. A medium dose of hypervirulent *Mtb* HN878 infection caused Lyl1^-/-^ mice to succumb to infection as early as 12-weeks post-infection compared to 24-weeks for WT mice (**Figure 4A**). A follow-up of mortality study revealed significant susceptibility to TB in Lyl1^-/-^ mice. The strong phenotype was translated to increased mycobacterial burden in Lyl1-deficient lung and spleen (**Figure 4B** *)* accompanied by increased organ weight index (**Figure 4C**) at chronic stages of 6- and 10-weeks post-infection but not earlier stages of 3-weeks post-infection.

It is known that *Mtb* bacterial growth can induce exacerbated lung pathology with an undesired and ineffective inflammatory response to the lungs that results in severe lung damage (35). Therefore, histopathological analysis using H&E staining to determine lung pathology was performed on WT and Lyl1^-/-^ lungs (**Figure 4D**). With the increased mycobacterial burden in the lungs of Lyl1^-/-^ mice, increased pathology, and inflammation, especially at the chronic stage of 10-weeks post-infection were observed. The phenomenon of exacerbated, uncontrolled lung damage was directly proportional to the level of iNOS and myeloperoxidase (MPO) present in the highly inflamed lungs, where a significant increase in iNOS and MPO levels in Lyl1-deficient mouse lungs was noted compared to WT mice (**Figures 4E, F**).

Together, the data demonstrate host susceptibility in the absence of Lyl1 in response to chronic hypervirulent *Mtb* HN878 infection. Additionally, Lyl1 deficiency resulted in susceptibility to the *Lm* infection (**Supplementary Figure 1H**). Overall, increased bacterial burdens and exacerbated inflammation in the site of infection can drive susceptibility, yielding Lyl1^-/-^ mice to succumb to the *Mtb* infection.

### The absence of Lyl1 increases neutrophil recruitment as well as chemokine secretion in response to hypervirulent *Mtb* HN878 infection *in vivo*

**Figure 5:**
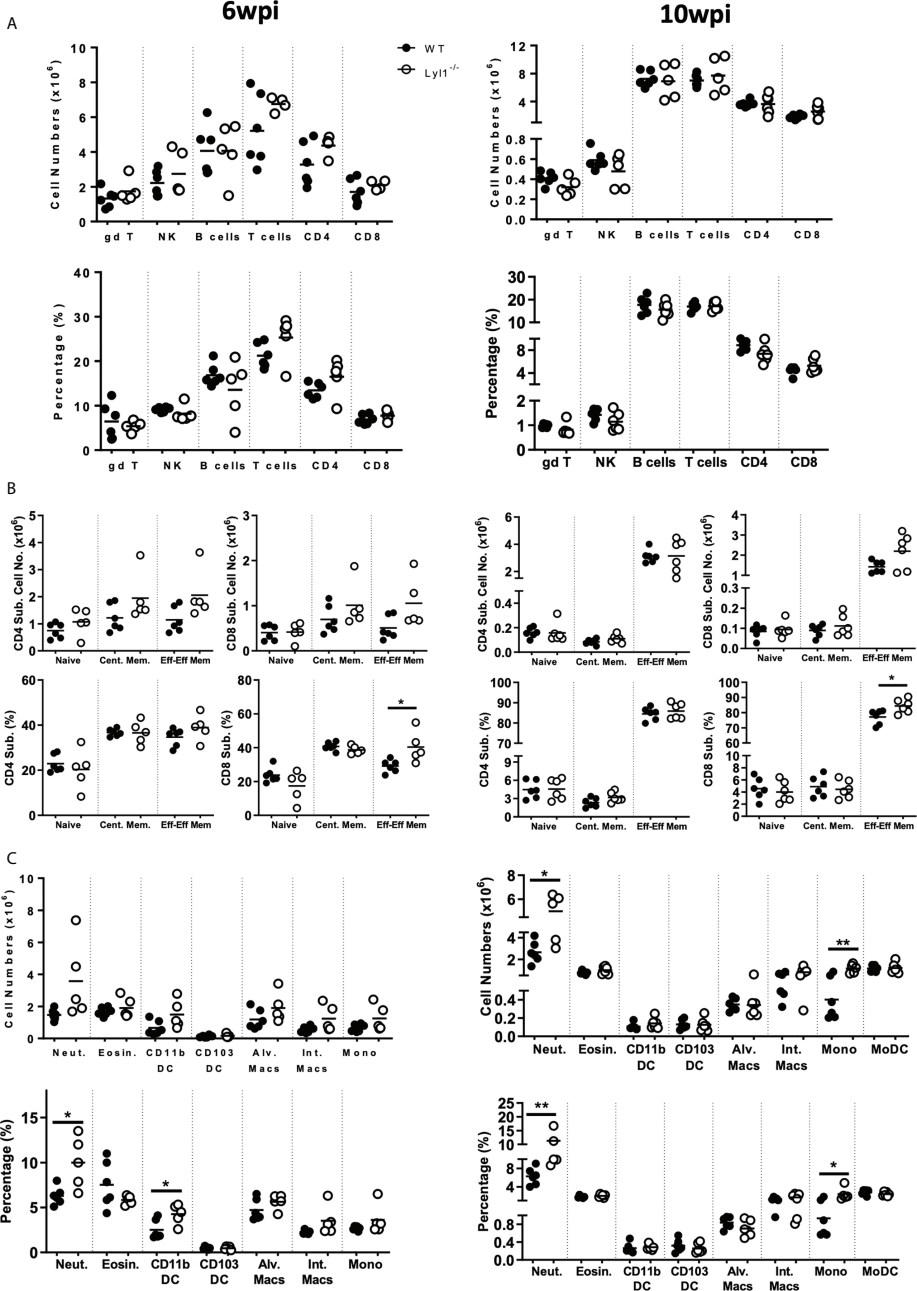
Lyl1-deletion enhances lung neutrophil and monocyte recruitment in response to chronic hypervirulent Mtb infection *in vivo*. **(A-C)** Littermate control (WT) and Lyl1^-/-^ mice were infected with ~100 CFU/mouse intranasally with Mtb HN878 (n = 5 mice/group) and sacrificed at either 6- or 10-weeks post infection. Using flow cytometry, single cell suspension of lung tissue was analyzed for **(A, B)** lymphoid and **(C)** myeloid total cell numbers and percentages of live cells. Naïve, central memory and effector/effector memory percentages are presented as ratio in the parent CD4 or CD8 population. Surface markers of the different cell populations are as follows (according to the gating strategy Figures S5-10): gamma delta T-cells (gd T) = CD3^+^gdTCR^+^; NK cells = NK1.1^+^CD3^-^; B-cells = CD19^+^CD3^-^; T-cells = CD3^+^CD19^-^; CD4^+^ T-cells = CD3^+^CD4^+^; CD8^+^ T-cells = CD3^+^CD8^+;^ Neutrophils (Neut.) = Ly6G^+^CD11b^+^; Eosinophils (Eosin.) = SiglecF^+^CD11b^+^CD64^-^; CD11b^+^ DC = CD11c^+^MHCII^+^CD11b^+^CD64^-^; CD103^+^ DC = CD11c^+^MHCII^+^CD103^+^CD64^-^; Alveolar Macrophages (Alv. Macs) = CD64^+^MerTK^+^SiglecF^+^CD11c^+^; Interstitial Macrophages (Int. Macs) = CD64^+^MerTK^+^SiglecF^-^CD11b^+^ CD11c^-^; Monocytes (Mono) = Ly6C^+^CD11b^+^CD64^-^; Monocyte-derived DC (MoDC) = CD64^+^CD11b^+^CD11c^+^. Line denotes Mean. Data shown is representative of 2-3 independent experiments. Unpaired student t-test analysis at *p < 0.05, **p < 0.01 to determine significance.

**Figure 6:**
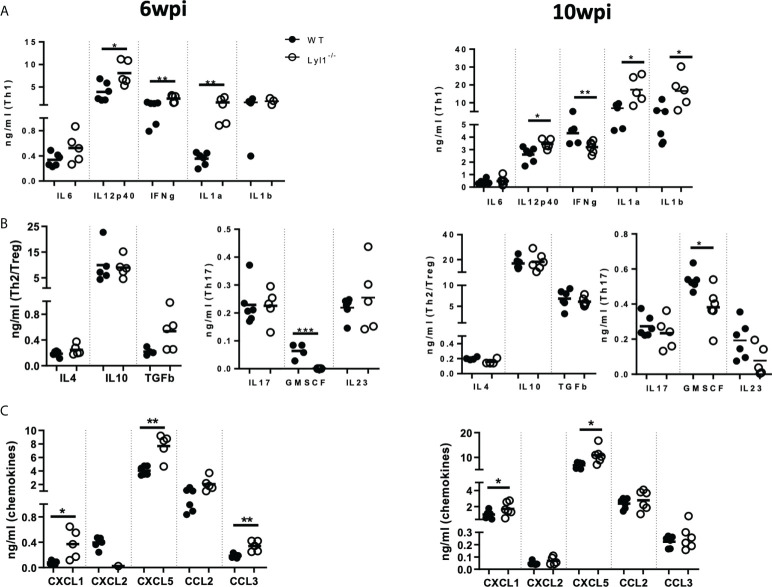
Lyl1-deletion promotes inflammatory responses during chronic hypervirulent *Mtb in vivo*. **(A-C)** Littermate control (WT) and Lyl1^-/-^ mice were infected with 100 CFU/mouse intranasally with *Mtb* HN878 (*n =* 5 mice/group) and sacrificed at either 6- or 10-weeks post-infection. Supernatants from lung homogenates were analyzed by ELISA for **(A)** Th1, **(B)** Th2 and T-regulatory cytokines, Th17 cytokines, and growth factors **(C)** and chemokines. Line denotes Mean. Data shown are representative of 2-3 independent experiments. Unpaired student t-test analysis at *p < 0.05, **p < 0.01 to determine significance.

Since Lyl1-deficiency causes increased mycobacterial burden and host susceptibility in response to *Mtb* HN878 infection, we aimed to investigate the differences in cellular recruitment patterns between WT and Lyl1^-/-^ mice to highlight the potential causes or consequences of host susceptibility in Lyl1-deficient mice. Lyl1 has been reported to play a significant role in T- and B-cell development (5) and the observed phenotype of susceptibility against *Mtb* infection has been noted to be pronounced during chronic infection where adaptive immunity to TB plays a fundamental role. Therefore, flow cytometric analysis of the distribution of lymphoid cells in the lungs (**Figures 5A, B**) of WT and Lyl1^-/-^ mice was investigated during the chronic stage of *Mtb* infection. Surprisingly, the lymphoid population in Lyl1-deficient *Mtb*-infected lungs was comparable to that of WT. However, an increase in neutrophils and monocytes was observed in 10-week *Mtb*-infected Lyl1^-/-^ lungs compared to WT (**Figure 5C**). This was later supported by various cytokines, and chemoattractants, including a significant increase in IL-12p40 and IL-1α at 6- and 10-weeks post-infection (**Figure 6A**). A contrasting expression in IFN-γ was observed whereby an increase at 6-weeks and a decrease at 10-weeks infected Ly1l-deficient lungs (**Figure 6A**). Furthermore, Lyl1^-/-^ lungs exhibited reduced GM-CSF levels at both 6- and 10-weeks post-*Mtb* HN878 infection (**Figure 6C**). Moreover, the secretion of neutrophil chemoattractants, CXCL1 and CXCL5, were significantly induced after 6- and 10-weeks post-infection in the absence of Lyl1 (**Figure 6B**).

Collectively, the data suggest that although Lyl1 is important for the early development of various components of the lymphoid population, the absence of this transcription factor does not affect the recruitment and effector-memory phenotype of these cells to the site of infection in response to hypervirulent *Mtb* HN878. However, the loss of Lyl1 does indeed induce neutrophil and monocyte recruitment to the site of infection, as well as excessive neutrophil chemo-attractant secretion, which could potentially be compromising the immune system for the exaggerated inflammation and effective eradication of *Mtb* HN878.

## Discussion

This study aims to uncover an under-appreciated role for Lyl1 under various immune stimuli. Since Lyl1 was originally discovered upon ectopic expression during T-ALL (3), downstream investigations focused on its oncogenic potential (6). However, we demonstrate novel functions of Lyl1 during bacterial infection in both *in vitro* and *in vivo* murine models. Host susceptibility to both *Mtb* HN878 and *Lm* in the absence of Lyl1 substantiates the host protective potential of this transcription factor. Furthermore, increased *Mtb* CFU in Lyl1^-/-^ macrophages and mice demonstrate a demand for Lyl1 regulated pathways to circumvent *Mtb* infection.

We show downregulation of Lyl1 expression after *Mtb* infection and report a host regulative mechanism rather than immune evasion. We also support this by proving Lyl1 regulation by MAPk and NF-κB signaling. Therefore, the unexpected host susceptibility in Lyl1^-/-^ mice suggests that Lyl1-associated pathways could be implicated in TB protection. During chronic stages, Lyl1^-/-^ mice demonstrate differential neutrophil recruitment and proinflammatory cytokine and chemokine secretion in comparison to WT hosts. Although IL-1 is known to provide immunity against *Mtb* infection (36, 37), unregulated IL-1 levels can also drive pathology during *Mtb* infection (38, 39). Additionally, increased IL-1 levels result in the recruitment of disease-promoting neutrophils, and polymorphisms associated with high IL-1 expression correlate with increased neutrophils in the bronchoalveolar lavage fluid of active TB patients (40, 41). Furthermore, interferons have also been described as key regulators of IL-1 (37, 38, 42). Here, we demonstrate differential expression of IFN-γ and IL-1. At 10-weeks post-infection, Lyl1^-/-^ lungs reveal decreased IFN-γ with increased IL-1 levels in comparison to WT lungs. Moreover, neutrophilic inflammation with supporting increased chemokine secretion in Lyl1-deficient lungs could promote *Mtb* susceptibility by inducing tissue lung damage as previously reported (43). Neutrophilic inflammation is further supported by an increase in histopathological iNOS and myeloperoxidase (MPO) as neutrophils are known for their ability to produce nitrogen intermediates as well as myeloperoxidase to limit pathogen survival and dissemination. However, the increased iNOS protein observed by immunohistochemistry staining could rather be a consequence of increased bacterial burden in Lyl1^-/-^ lungs since macrophage-specific NOS2 is significantly downregulated after *Mtb* infection in the absence of Lyl1. Therefore, since NOS2 functions in the clearance of *Mtb* (44), possibly through apoptosis (45), reduced macrophage-specific NOS2 in Lyl1^-/-^ macrophages could contribute to increased bacterial burden and inflammation in the lungs. Previous reports have demonstrated a distinct relationship between GM-CSF, alveolar macrophages, and bacterial clearance in the lungs (46, 47). In the absence of GM-CSF, macrophages become more permissive to *Mtb* growth (47, 48) as GM-CSF has been shown to play a role in the proliferation, maintenance, and function of alveolar macrophages (46, 49). The reduction in GM-CSF levels in Lyl1-deficient lungs could result in defective alveolar macrophage function and thus encourage *Mtb* growth. The pleiotropic transcription factor, Lyl1 was also shown to play role in adult angiogenesis. Tumor cells implanted in Lyl1-deficient mice grew faster with increased permeability of tumor vasculature (50). Increased vascular permeability and angiogenesis are characteristics of tuberculous granulomas and normalization of granuloma vasculature by vascular endothelial growth factor (VEGF) inhibition can increase anti-TB drug delivery, excessive granulomatous inflammation and dissemination from the lungs (51–53).

In summary, we provide evidence to support novel, non-leukemia-associated functions of Lyl1 during bacterial infections. Our study is limited by exploring macrophage-specific roles of Lyl1 in *Mtb* infection to understand and delineate the mechanisms behind the susceptibility of Lyl1^-/-^ mice. Existing mouse single-cell transcriptomics data show that Lyl1 is highly expressed in lung monocytes-macrophages and observed phenotypes in chronic *Mtb* infected mice are more myeloid-centric rather than lymphoid-centric. These observations hint that deletion of Lyl1 in macrophage subsets has more deleterious effects than lymphoid cells in adult mice in response to bacterial infections. However; our findings warrant further research into cell specific or inducible Lyl1 knockout mouse models to delineate the role of the intriguing transcription factor in infection disease models. Since Lyl1 expression was comparable in TB patients and healthy individuals, the expression of Lyl1 cannot be used as a biomarker for TB in whole blood. Instead, differential expression of Lyl1 in whole blood was present in acute systemic viral infections, suggesting a further role for Lyl1 in response to viral pathogens as an early response gene. Therefore, this study reveals an underestimated role for Lyl1 in various immune responses. Although Lyl1-associated pathways further need to be investigated, our data highlights important immune regulatory functions of Lyl1 during Mtb infection.

## Data availability statement

The original contributions presented in the study are included in the article/**Supplementary Material**. Further inquiries can be directed to the corresponding authors.

## Ethics statement

The studies involving human participants were reviewed and approved by Human Ethics Committee Faculty of Health Sciences, University of Cape Town, Cape Town (HREC Ref Number: 140 732/2015). Written informed consent for participation was not required for this study in accordance with the national legislation and the institutional requirements. The animal study was reviewed and approved by Animal Research Ethics Committee of the Faculty of Health Sciences, University of Cape Town (Protocol Permit No: 015/040 and 019/023).

## Author contributions

S-SJ, MO, FB, and RG contributed to the study conception and design. Material preparation, data collection, and analysis were performed by S-SJ, MO, NK, SKLP, RH, and OT. SP, HS, FB, and RG conceived and managed the research. The first draft of the manuscript was written by S-SJ and all authors commented on previous versions of the manuscript. All authors contributed to the article and approved the submitted version.

## Funding

This work was supported by the Department of Science and Technology (DST)/South African National Research Foundation (NRF) PhD fellowship and Carnegie Corporation Postdoctoral Fellowship to S-SJ; ICGEB Arturo Falaschi postdoctoral fellowship and EDCTP grant-holder linked postdoctoral fellowship to MO; SAMRC Internship Scholarship Programme, Division of Research Capacity Development (RCD) to SKLP; WUN CIDRI-Africa PhD fellowship to RH; Research Grant For the Special Coordination Funds for Promoting Science and Technology from the Ministry of Education, Culture, Sports, Science and Technology of the Japanese Government (MEXT) to HS; the grants from the NRF/DST-South African Research Chair Initiative (SARCHi), South Africa Medical Research Council (SAMRC) and the International Centre for Genetic Engineering & Biotechnology (ICGEB) to FB; NRF Competitive Programme for Unrated Researchers (CSUR), the DST/NRF Collaborative Postgraduate Training Programme as well as the BRICS Multilateral Joint Science and Technology Research Collaboration grant number 110482 to RG. This research was funded in whole, or in part, by the Wellcome Trust [203135/Z/16/Z]. For the purpose of open access, the author has applied a CC BY public copyright license to any Author Accepted Manuscript version arising from this submission. The funders had no role in the study design, data collection, and analysis, decision to publish, or preparation of the manuscript.

## Acknowledgments

We express gratitude to the UCT Animal Research Facility and Ms. Munadia Ansarie for the breeding, genotyping, and maintenance of mice as well as the technical staff. Ms. Zarinah Sonday, Mr. Marlon Petersen, and Mr. George Jacobs for the maintenance of the laboratory. Furthermore, we thank Ms. Lizette Fick and Ms. Raygaana Jacobs for their excellent histological services.

## Conflict of interest

The authors declare that the research was conducted in the absence of any commercial or financial relationships that could be construed as a potential conflict of interest.

## Publisher’s note

All claims expressed in this article are solely those of the authors and do not necessarily represent those of their affiliated organizations, or those of the publisher, the editors and the reviewers. Any product that may be evaluated in this article, or claim that may be made by its manufacturer, is not guaranteed or endorsed by the publisher.

## Author’s disclaimer

The content hereof is the sole responsibility of the authors and does not necessarily represent the official views of the funders.

## Supplementary material

Supplementary Figure 1 Human macrophage LYL1 is downregulated in response to bacterial infections and immune stimulants as well as Lyl1 deletion renders mice more susceptible to Lm infection. **(A)** Expression kinetics (represented as Tags Per Million (TPM)) of Lyl1 in uninfected mouse bone marrow-derived macrophage (BMDM) data were extracted from the FANTOM5 mouse macrophages dataset. **(B)** Methylthialazole Tetrazolium (MTT) assay performed in differentially polarized Mtb HN878 infected BMDM at 48 hours and 72 hours post infection. The expression kinetics (represented as Tags Per Million (TPM)) of LYL1 in Mtb HN878 infected **(C)** unstimulated Mtb HN878 infected human monocyte-derived macrophage (MDM) dataset and **(D)** LPS-stimulated human monocyte-derived macrophage (MDM) data were extracted from the FANTOM5 dataset. **(E)** C57BL/6 mice were administered either 10 mg/kg LPS or PBS by intraperitoneal injection (n = 6 mice/group) after which indicated organs were collected at 4- and 8-hours post-administration for RNA isolation to measure Lyl1 mRNA expression by RT-qPCR. **(F)** Wild type mice were infected with ~100 CFU/mouse intranasally with Mtb HN878 (n = 5-6 mice/group) and sacrificed at 3-, 6- or 10- weeks post-infection. RNA isolation was performed on lung homogenates. RT-qPCR was performed on synthesized cDNA to investigate Lyl1 mRNA expression. **(G)** Single-cell transcriptomics of murine lungs extracted from the Tabula Muris database showing Lyl1 expression in different lung cell types. Scale, ln (1+counts per million, CPM). **(H)** A survival study (n = 10-17 mice/group) by intraperitoneally injecting WT, Lyl1^+/-^, and Lyl1^-/-^ mice with 1.9x10^6^ CFU/mouse Lm. Mantel-Cox survival analysis with log-rank test P = 0.0097, WT vs. Lyl1^-/-^. Error bar denotes Mean ± SEM. Data shown are representative of 2-4 independent experiments. Unpaired student t-test analysis at *p < 0.05, **p < 0.01, ***p < 0.001, ****p < 0.0001 to determine significance.

Supplementary Figure 2 Lyl1 expression is downregulated in acute viral infections in contrast to chronic TB cohort. All represented data was extracted from publicly available databases. **(A)** Data were manually extracted, organized, and sorted from the publicly available TB Gambia Cohort (PMID 22046420). **(B)** Data were manually extracted, organized, and sorted from the publicly available TB South Africa (SA) and United Kingdom (UK) Cohort (PMID 20725040). **(C)** Data were manually extracted, organized, and sorted from the publicly available USA Rhinovirus and Influenza A Cohort (PMID 26070066). Kruskal-Wallis test analysis at **p <* 0.05, ***p <* 0.01, ****p <* 0.001, *****p <* 0.0001 to determine significance.

Supplementary Figure 3 Confirmed globally deleted Lyl1 mice do not induce changes in cell recruitment and pathology in the lung at the steady state. Naïve control littermates (WT) and Lyl1^-/-^ mice (n = 5-6 mice/group) were euthanized and investigated at homeostasis. **(A)** Global deletion of Lyl1 was confirmed by RT-qPCR, relative to Hprt housekeeping gene, on isolated mRNA from indicated tissues. **(B)** Absolute cell numbers in the liver, lung, lymph node (LN), spleen and thymus. **(C)** Flow cytometry was used to determine absolute numbers of lung Ly6G^+^CD11b^+^ neutrophils (Neut.); SiglecF^+^CD11b^+^CD64^-^ Eosinophils (Eosin.); CD11c^+^MHCII^+^CD11b^+^CD64^-^ DC; CD11c^+^MHCII^+^CD103^+^CD64^-^ DC; CD64^+^MerTK^+^SiglecF^+^CD11c^+^ Alveolar Macrophages (Alv. Macs); CD64^+^MerTK^+^SiglecF^-^CD11b^+^ CD11c^-^ Interstitial Macrophages (Int. Macs); Ly6C^+^CD11b^+^CD64^-^ Monocytes (Mono); CD64^+^CD11b^+^CD11c^+^ Monocyte-derived DC (MoDC); **(D)** CD3^+^gdTCR^+^ gamma delta T-cells (gd T); NK1.1^+^CD3^-^ NK cells; CD19^+^CD3^-^ B-cells; CD3^+^CD19^-^ T-cells; CD3^+^CD4^+^ T-cells; CD3^+^CD8^+^ T-cells; **(E)** CD3^+^CD4^+^CD62L^+^CD44^-^ naïve; CD3^+^CD4^+^CD62L^+^CD44^+^ Central Memory; CD3^+^CD4^+^CD44^+^CD62L^-^ Effector/Effector Memory; CD3^+^CD8^+^CD62L^+^CD44^-^ naïve; CD3^+^CD8^+^CD62L^+^CD44^+^ Central Memory; CD3^+^CD8^+^CD44^+^ CD62L^-^ Effector/Effector Memory. **(F)** Representative histopathology sections (x20 magnification) for H&E (scale bar = 1000 µm) with **(G)** quantified alveolar spaces from 1-2 deep cut lung sections per mouse (30 µm apart). Data shown are representative of two independent experiments. Line denotes Mean. Unpaired student’s t-test was used to confirm insignificant differences between the two genotypes.

Supplementary Figure 4 Global deletion of Lyl1 minor effects on cell recruitment to the lymph node, spleen, and liver with decreased T cell maturation on DN stage in the thymus. Indicated organs from both WT and Lyl1^-/-^ naïve mice (n = 6 mice/group) were harvested and processed for single-cell suspension for downstream flow cytometric analysis investigating the lymphoid and myeloid populations as per the gating strategies outlined in Figures S5-10. This included the lymph node **(A–C)** lymphoid and **(D)** myeloid population, the **(E)** thymus lymphoid population, the spleen **(F)** myeloid and **(G, H)** lymphoid population as well as the liver **(I)** myeloid and **(J–L)** lymphoid population. Line denotes Mean. Data shown are representative of two independent experiments. Unpaired student t-test analysis at *p < 0.05, **p < 0.01 to determine significance.

Supplementary Figure 5 Gating strategy to determine the lymphoid cell populations in the lung, lymph node, spleen, and liver.

Supplementary Figure 6 Gating strategy to determine the myeloid cell populations in the lung.

Supplementary Figure 7 Gating strategy to determine the lymphoid cell populations in the thymus.

Supplementary Figure 8 Gating strategy to determine the myeloid cell populations in the liver.

Supplementary Figure 9 Gating strategy to determine the myeloid cell populations in the lymph node.

Supplementary Figure 10 Gating strategy to determine the myeloid cell populations in the spleen.

Supplementary Figure 11 Representative Ziehl-Neelsen immunohistochemistry lung section (x1000 magnification) with Mtb positive areas indicated by arrows at 3, 6 and 10 weeks post infection.

Supplementary Table 1 List of all the primer sequences.
