# Supplementary material for: Lyl1-deficiency promotes inflammatory responses and increases mycobacterial burden in response to Mycobacterium tuberculosis infection in mice
Source: Front Immunol. 2022 Sep 2;13:948047. doi: 10.3389/fimmu.2022.948047 (PMC9481033; doi:10.3389/fimmu.2022.948047)
Supplement: Supplementary Figure 1 — Human macrophage LYL1 is downregulated in response to bacterial infections and immune stimulants as well as Lyl1 deletion renders mice more susceptible to Lm infection. (A) Expression kinetics (represented as Tags Per Million (TPM)) of Lyl1 in uninfected mouse bone marrow-derived macrophage (BMDM) data were extracted from the FANTOM5 mouse macrophages dataset. (B) Methylthialazole Tetrazolium (MTT) assay performed in differentially polarized Mtb HN878 infected BMDM at 48 hours and 72 hours post infection. The expression kinetics (represented as Tags Per Million (TPM)) of LYL1 in Mtb HN878 infected (C) unstimulated Mtb HN878 infected human monocyte-derived macrophage (MDM) dataset and (D) LPS-stimulated human monocyte-derived macrophage (MDM) data were extracted from the FANTOM5 dataset. (E) C57BL/6 mice were administered either 10 mg/kg LPS or PBS by intraperitoneal injection (n = 6 mice/group) after which indicated organs were collected at 4- and 8-hours post-administration for RNA isolation to measure Lyl1 mRNA expression by RT-qPCR. (F) Wild type mice were infected with ~100 CFU/mouse intranasally with Mtb HN878 (n = 5-6 mice/group) and sacrificed at 3-, 6- or 10- weeks post-infection. RNA isolation was performed on lung homogenates. RT-qPCR was performed on synthesized cDNA to investigate Lyl1 mRNA expression. (G) Single-cell transcriptomics of murine lungs extracted from the Tabula Muris database showing Lyl1 expression in different lung cell types. Scale, ln (1+counts per million, CPM). (H) A survival study (n = 10-17 mice/group) by intraperitoneally injecting WT, Lyl1+/-, and Lyl1-/- mice with 1.9x10^6^ CFU/mouse Lm. Mantel-Cox survival analysis with log-rank test P = 0.0097, WT vs. Lyl1-/-. Error bar denotes Mean ± SEM. Data shown are representative of 2-4 independent experiments. Unpaired student t-test analysis at *p < 0.05, **p < 0.01, ***p < 0.001, ****p < 0.0001 to determine significance. [file DataSheet_1.pdf]

Supplementary Figures

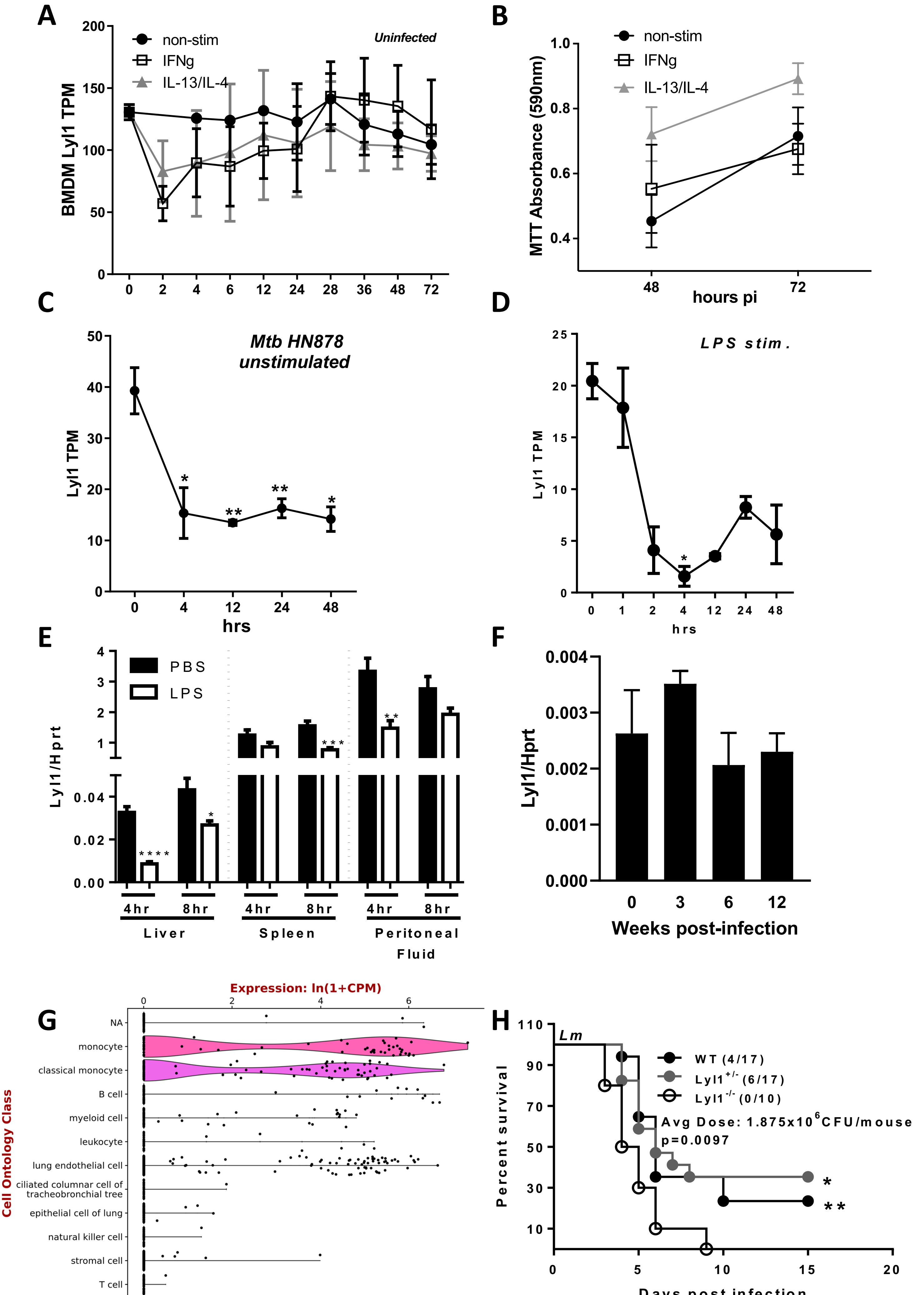
A
BMDM Lyl1 TPM
non-stim
IFNg
IL-13/IL-4
Uninfected
B
MTT Absorbance (590nm)
hours pi
non-stim
IFNg
IL-13/IL-4
C
Mtb HN878 unstimulated
Lyl1 TPM
hrs
D
LPS stim.
Lyl1 TPM
hrs
E
Lyl1/Hprt
PBS
LPS
4hr
8hr
Liver
Spleen
Peritoneal Fluid
F
Lyl1/Hprt
Weeks post-infection
G
Expression: ln(1+CPM)
Cell Ontology Class
NA
monocyte
classical monocyte
B cell
myeloid cell
leukocyte
lung endothelial cell
ciliated columnar cell of tracheobronchial tree
epithelial cell of lung
natural killer cell
stromal cell
T cell
H
Lm
WT (4/17)
Lyl1+/- (6/17)
Lyl1-/- (0/10)
Avg Dose: 1.875x10^6 CFU/mouse
p=0.0097
Percent survival
Days post infection

Figure S1

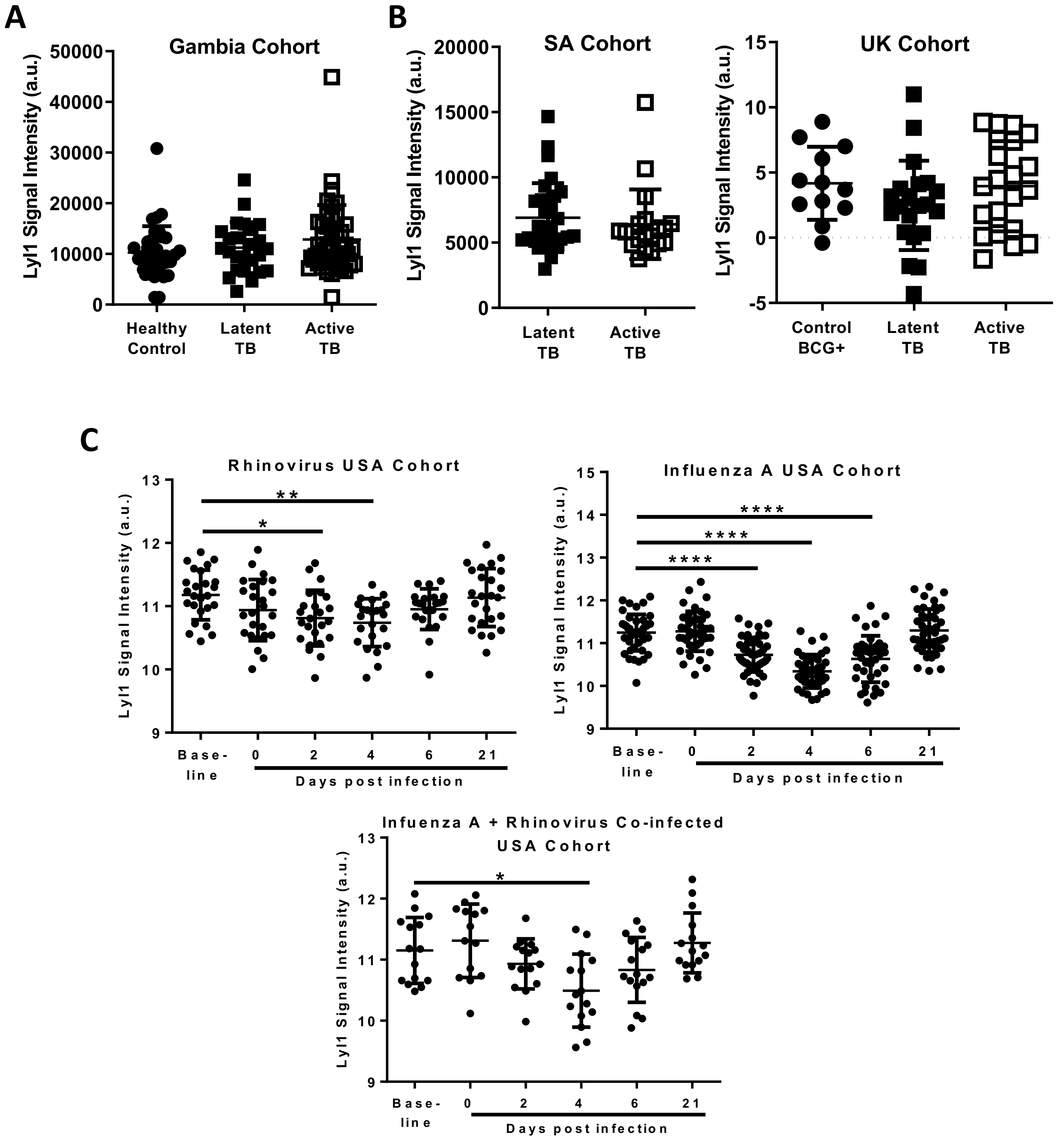
A
Gambia Cohort
Lyl1 Signal Intensity (a.u.)
50000
40000
30000
20000
10000
0
Healthy Control
Latent TB
Active TB
B
SA Cohort
Lyl1 Signal Intensity (a.u.)
20000
15000
10000
5000
0
Latent TB
Active TB
UK Cohort
Lyl1 Signal Intensity (a.u.)
15
10
5
0
-5
Control BCG+
Latent TB
Active TB
C
Rhinovirus USA Cohort
Lyl1 Signal Intensity (a.u.)
13
12
11
10
9
**
*
Base-line
0
2
4
6
21
Days post infection
Influenza A USA Cohort
Lyl1 Signal Intensity (a.u.)
15
14
13
12
11
10
9
****
****
****
Base-line
0
2
4
6
21
Days post infection
Infuenza A + Rhinovirus Co-infected USA Cohort
Lyl1 Signal Intensity (a.u.)
13
12
11
10
9
*
Base-line
0
2
4
6
21
Days post infection

Figure S2

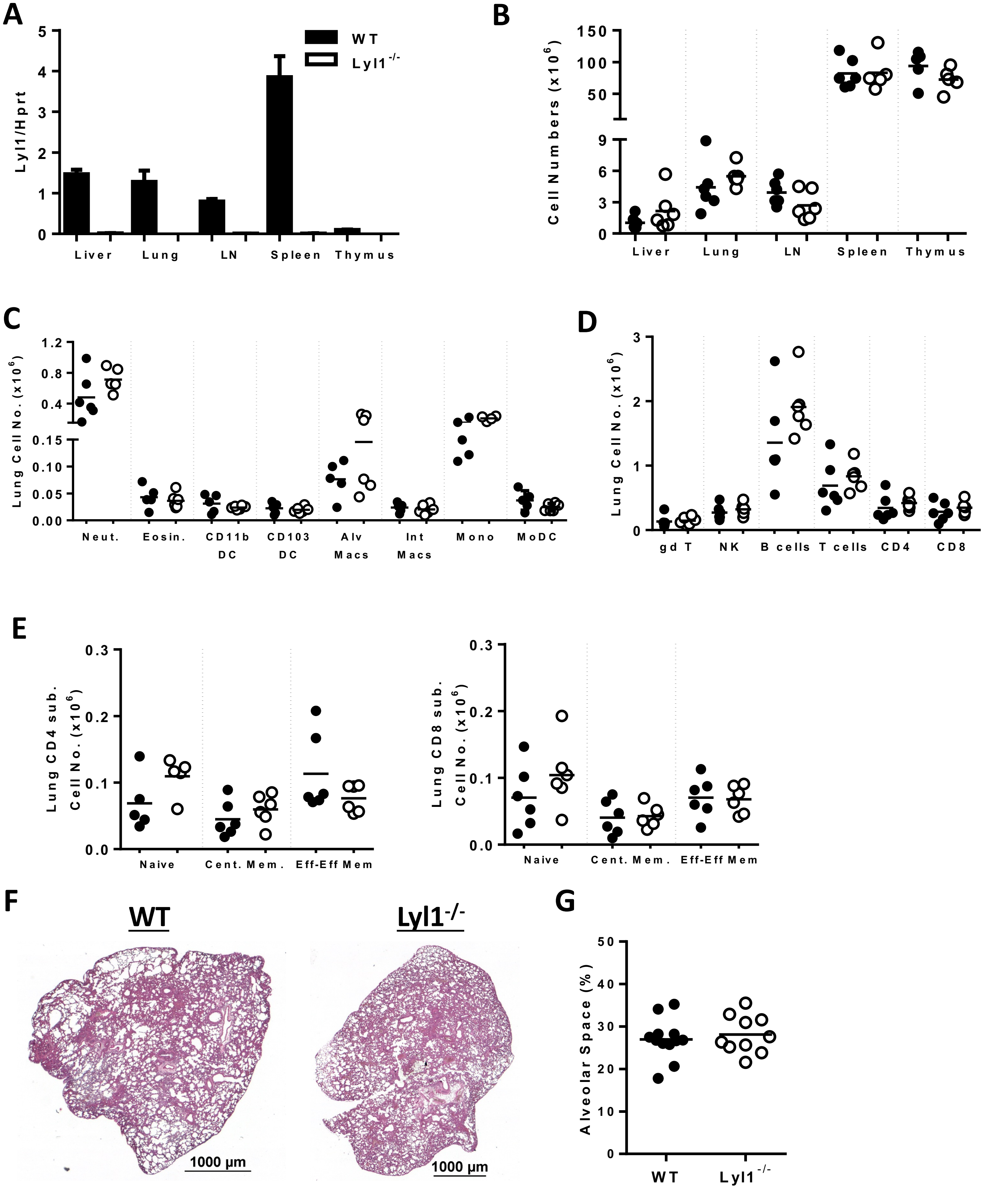
A
Lyl1/Hprt
WT
Lyl1-/-
Liver
Lung
LN
Spleen
Thymus
B
Cell Numbers (x10^6)
Liver
Lung
LN
Spleen
Thymus
C
Lung Cell No. (x10^6)
Neut.
Eosin.
CD11b DC
CD103 DC
Alv Macs
Int Macs
Mono
MoDC
D
Lung Cell No. (x10^6)
gd T
NK
B cells
T cells
CD4
CD8
E
Lung CD4 sub. Cell No. (x10^6)
Naive
Cent. Mem.
Eff-Eff Mem
Lung CD8 sub. Cell No. (x10^6)
Naive
Cent. Mem.
Eff-Eff Mem
F
WT
Lyl1-/-
1000 µm
1000 µm
G
Alveolar Space (%)
WT
Lyl1-/-

Figure S3

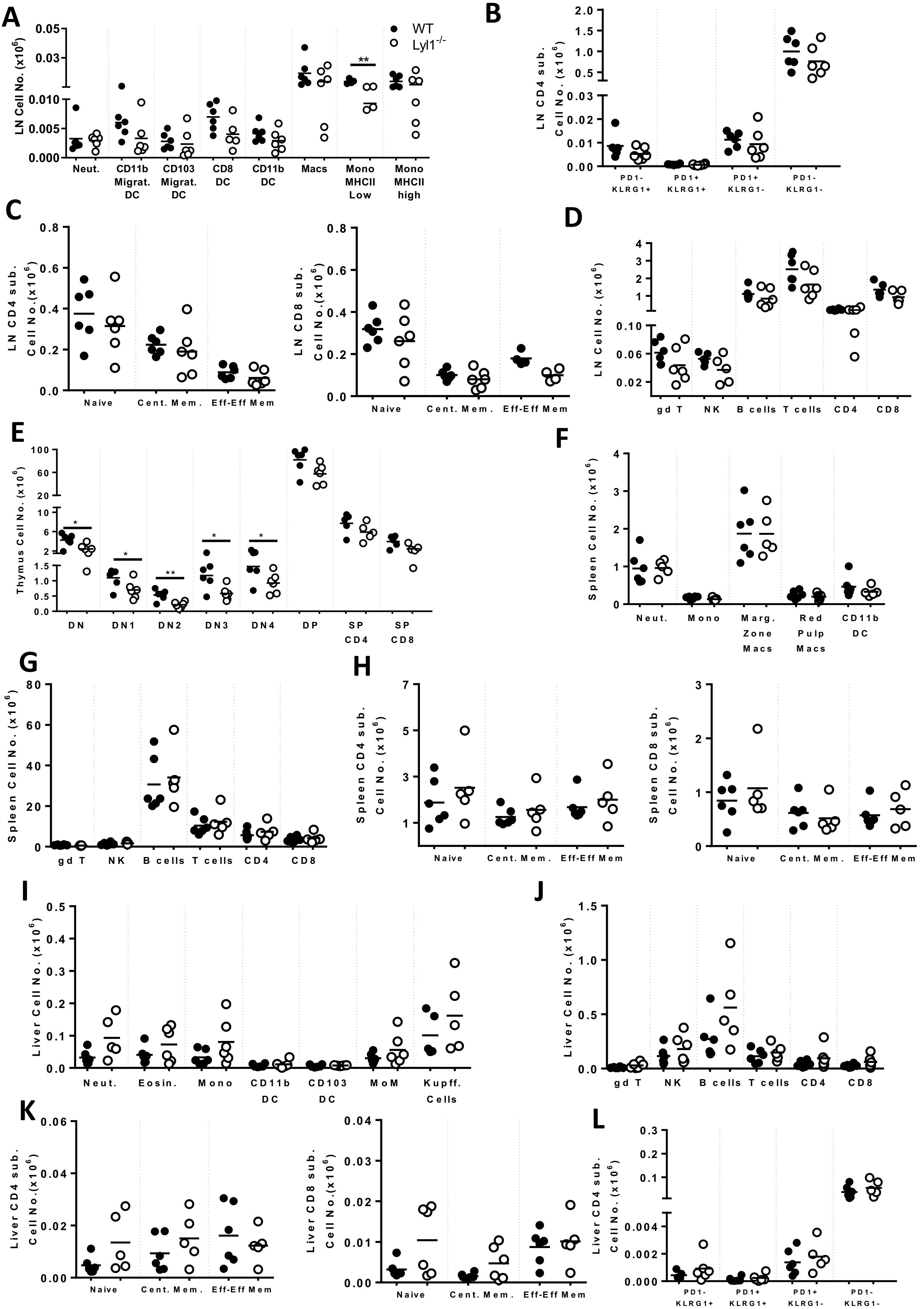
A
B
C
D
E
F
G
H
I
J
K
L
WT
Lyl1-/-
LN Cell No. (x10^6)
Neut.
CD11b Migrat. DC
CD103 Migrat. DC
CD8 DC
CD11b DC
Macs
Mono MHCII Low
Mono MHCII high
LN CD4 sub. Cell No. (x10^6)
PD1- KLRG1+
PD1+ KLRG1+
PD1+ KLRG1-
PD1- KLRG1-
LN CD8 sub. Cell No.(x10^6)
Naive
Cent. Mem.
Eff-Eff Mem
gd T
NK
B cells
T cells
CD4
CD8
Thymus Cell No. (x10^6)
DN
DN1
DN2
DN3
DN4
DP
SP CD4
SP CD8
Spleen Cell No. (x10^6)
Mono
Marg. Zone Macs
Red Pulp Macs
Spleen CD4 sub. Cell No. (x10^6)
Spleen CD8 sub. Cell No. (x10^6)
Liver Cell No. (x10^6)
Eosin.
CD103 DC
MoM
Kupff. Cells
Liver CD4 sub. Cell No.(x10^6)
Liver CD8 sub. Cell No.(x10^6)

Figure S4

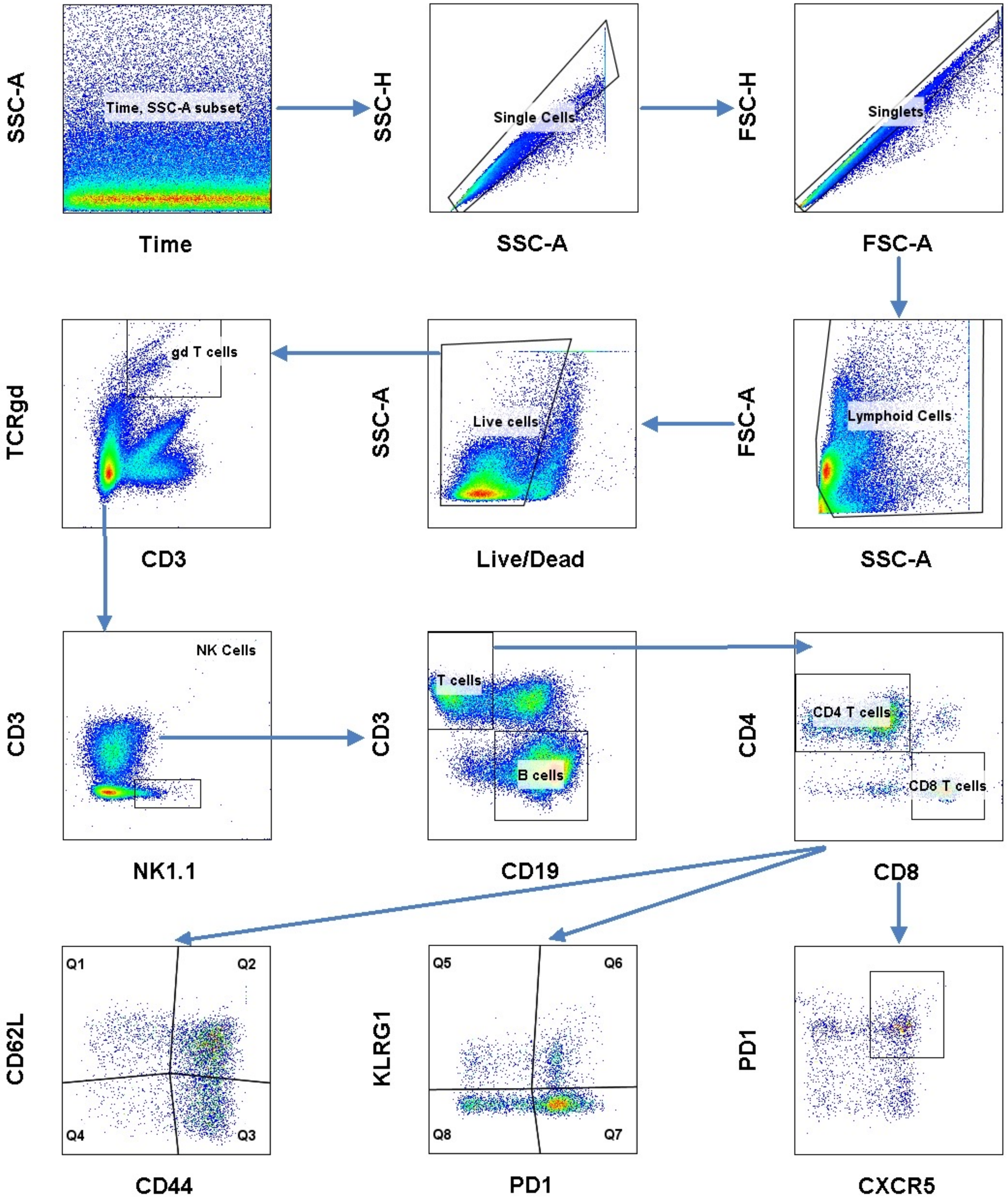

SSC-A
Time, SSC-A subset
Time
SSC-H
Single Cells
SSC-A
FSC-H
Singlets
FSC-A
TCRgd
gd T cells
CD3
SSC-A
Live cells
Live/Dead
FSC-A
Lymphoid Cells
SSC-A
CD3
NK Cells
NK1.1
CD3
T cells
B cells
CD19
CD4
CD4 T cells
CD8 T cells
CD8
CD62L
Q1
Q2
Q4
Q3
CD44
KLRG1
Q5
Q6
Q8
Q7
PD1
PD1
CXCR5

Figure S5

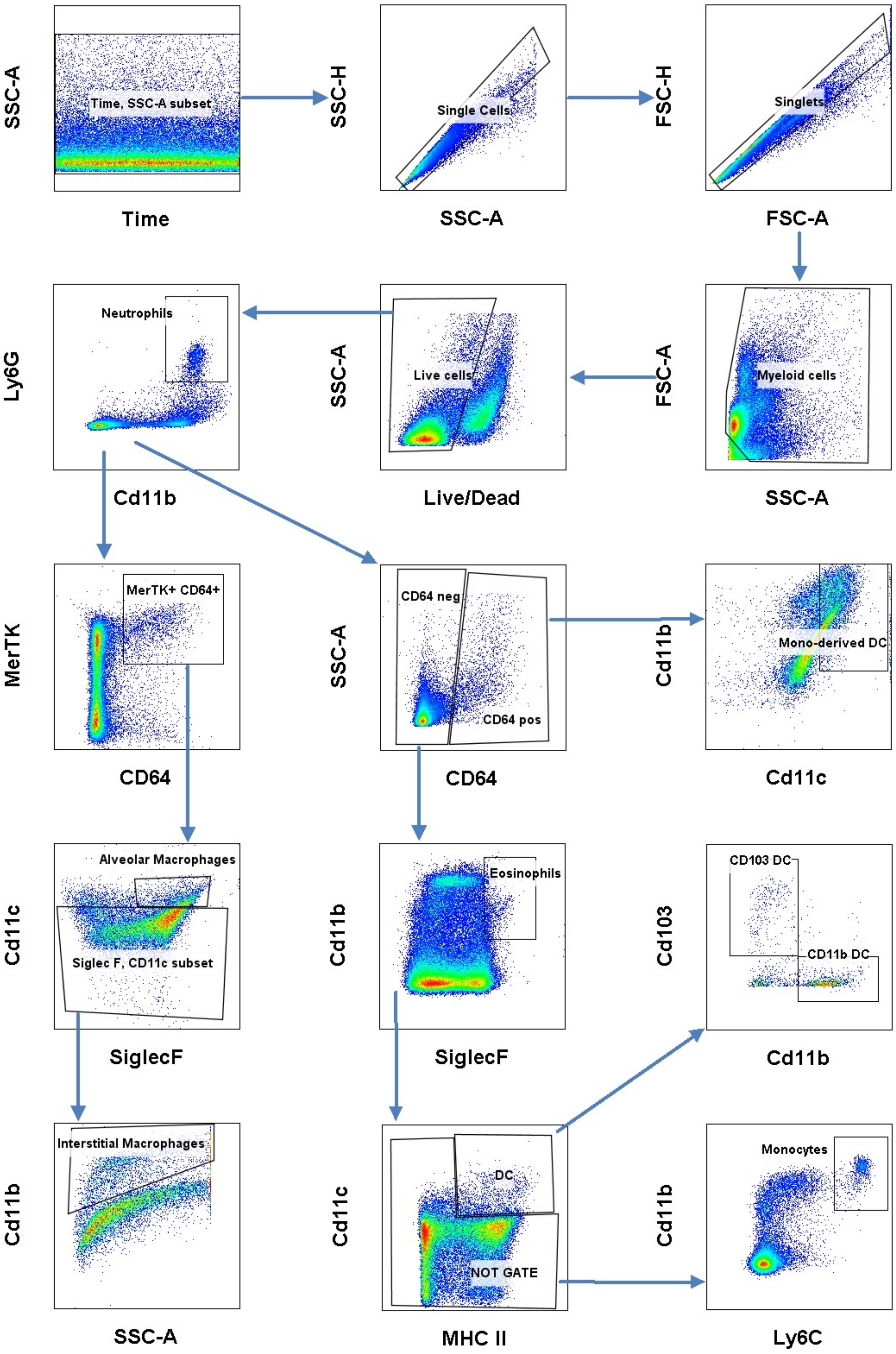
SSC-A
Time, SSC-A subset
Time
SSC-H
Single Cells
SSC-A
FSC-H
Singlets
FSC-A
Ly6G
Neutrophils
Cd11b
SSC-A
Live cells
Live/Dead
FSC-A
Myeloid cells
SSC-A
MerTK
MerTK+ CD64+
CD64
SSC-A
CD64 neg
CD64 pos
CD64
Cd11b
Mono-derived DC
Cd11c
Cd11c
Alveolar Macrophages
Siglec F, CD11c subset
SiglecF
Cd11b
Eosinophils
SiglecF
Cd103
CD103 DC
CD11b DC
Cd11b
Cd11b
Interstitial Macrophages
SSC-A
Cd11c
DC
NOT GATE
MHC II
Cd11b
Monocytes
Ly6C

Figure S6

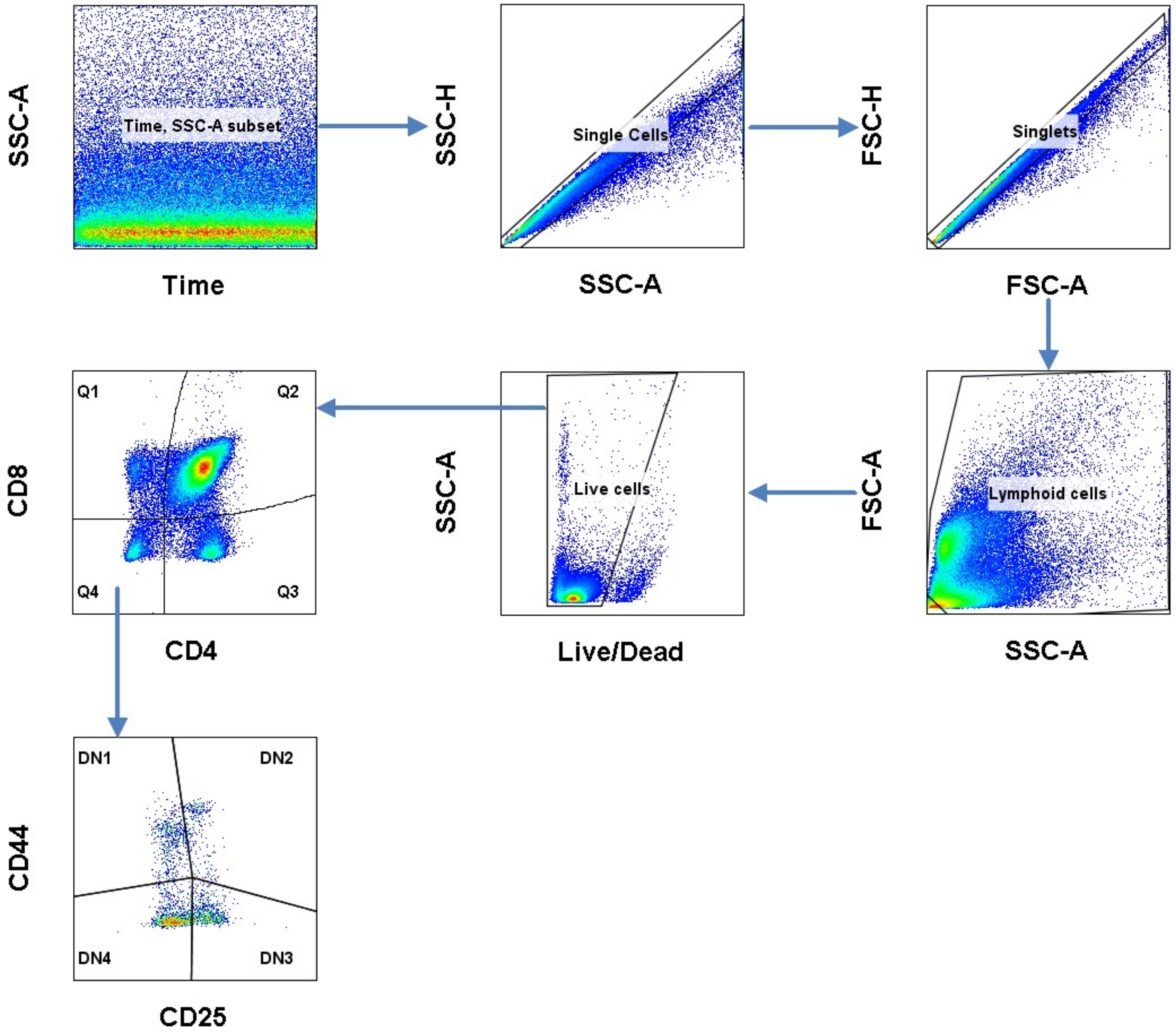
SSC-A
Time, SSC-A subset
Time
SSC-H
Single Cells
SSC-A
FSC-H
Singlets
FSC-A
FSC-A
Lymphoid cells
SSC-A
SSC-A
Live cells
Live/Dead
CD8
Q1
Q2
Q4
Q3
CD4
CD44
DN1
DN2
DN4
DN3
CD25

Figure S7

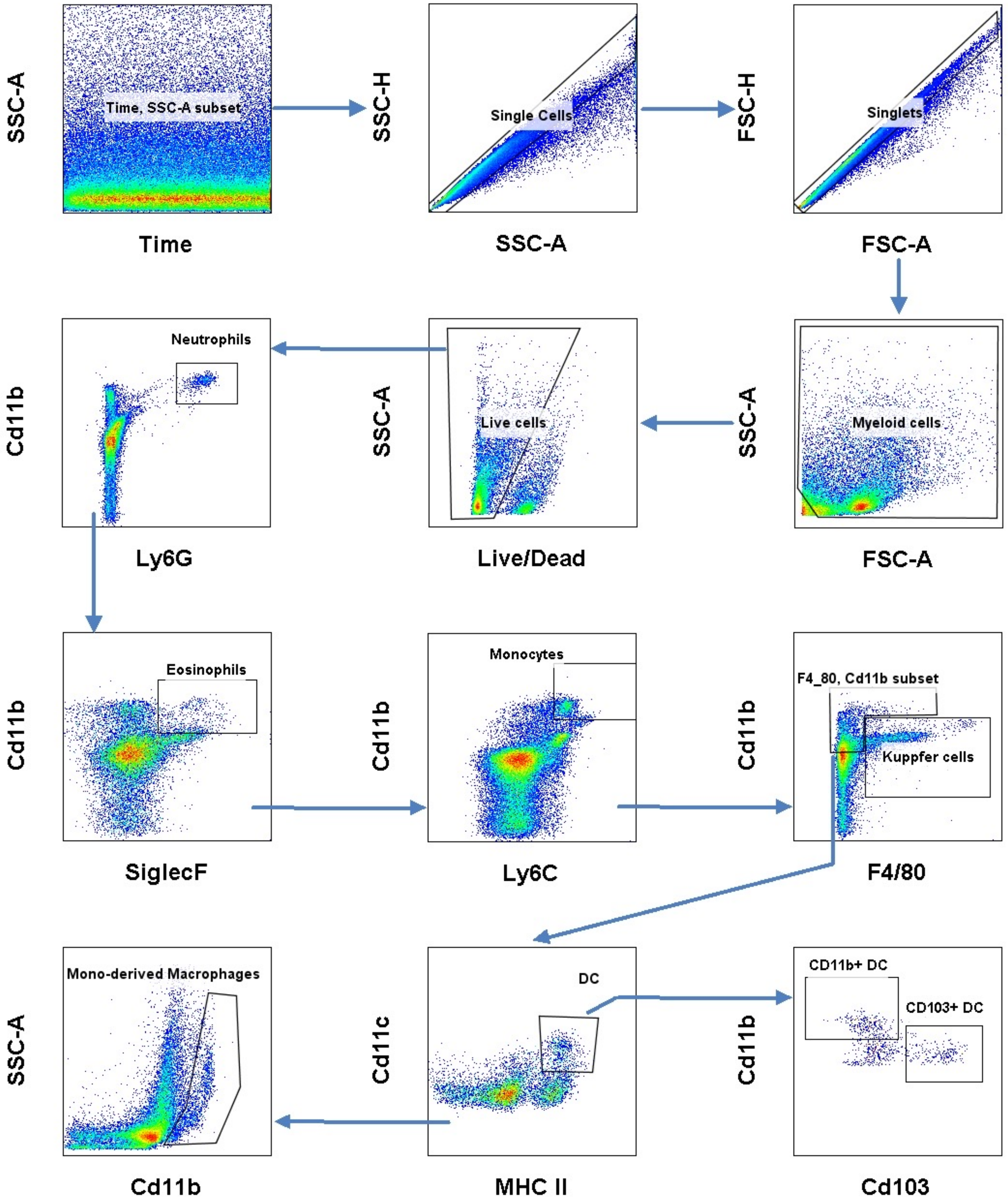
SSC-A
Time, SSC-A subset
Time
SSC-H
Single Cells
SSC-A
FSC-H
Singlets
FSC-A
Cd11b
Neutrophils
Ly6G
SSC-A
Live cells
Live/Dead
SSC-A
Myeloid cells
FSC-A
Cd11b
Eosinophils
SiglecF
Cd11b
Monocytes
Ly6C
Cd11b
F4_80, Cd11b subset
Kuppfer cells
F4/80
SSC-A
Mono-derived Macrophages
Cd11b
Cd11c
DC
MHC II
Cd11b
CD11b+ DC
CD103+ DC
Cd103

Figure S8

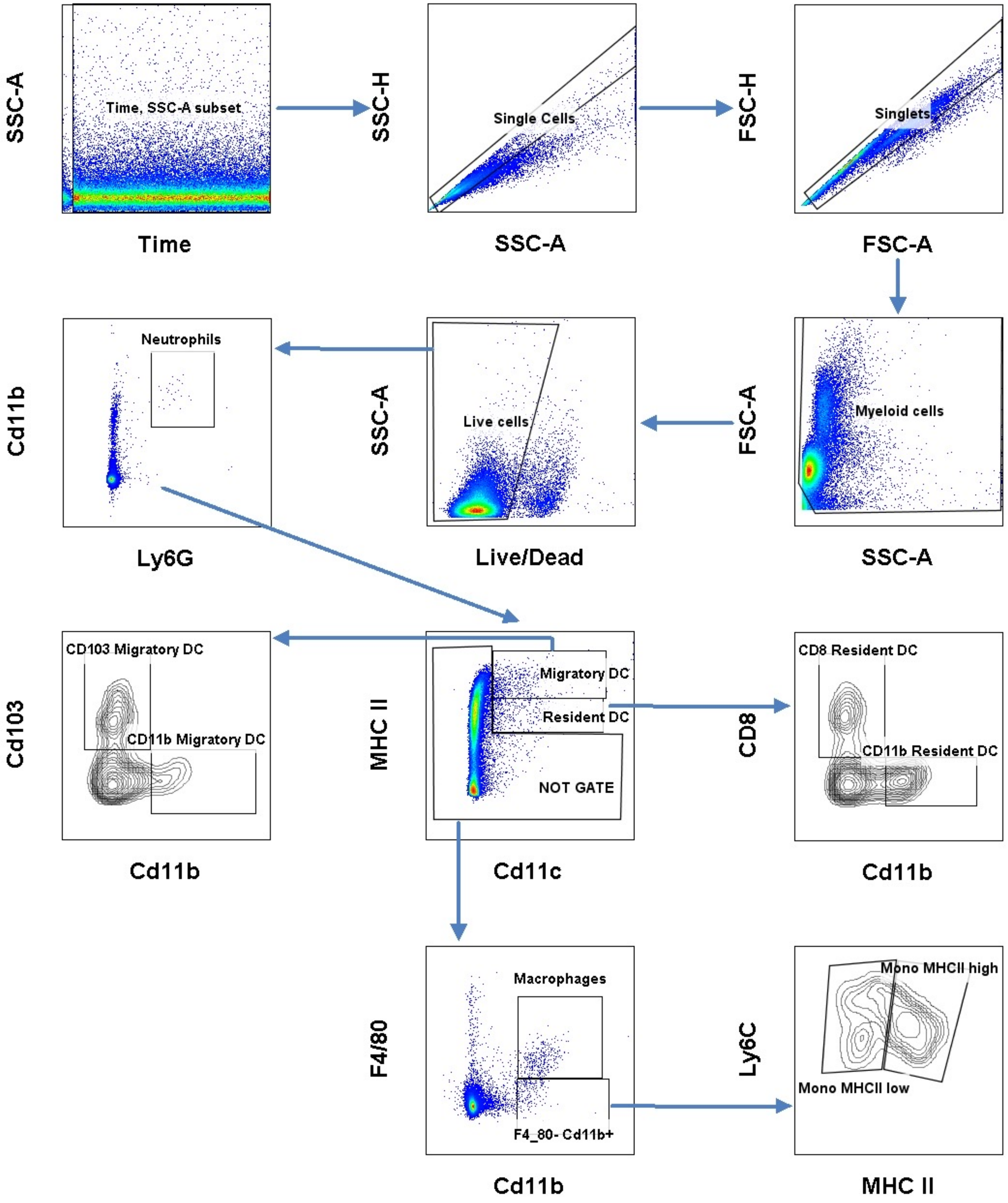

SSC-A
Time, SSC-A subset
Time
SSC-H
Single Cells
SSC-A
FSC-H
Singlets
FSC-A
Cd11b
Neutrophils
Ly6G
SSC-A
Live cells
Live/Dead
FSC-A
Myeloid cells
SSC-A
Cd103
CD103 Migratory DC
CD11b Migratory DC
Cd11b
MHC II
Migratory DC
Resident DC
NOT GATE
Cd11c
CD8
CD8 Resident DC
CD11b Resident DC
Cd11b
F4/80
Macrophages
F4_80- Cd11b+
Cd11b
Ly6C
Mono MHCII high
Mono MHCII low
MHC II

Figure S9

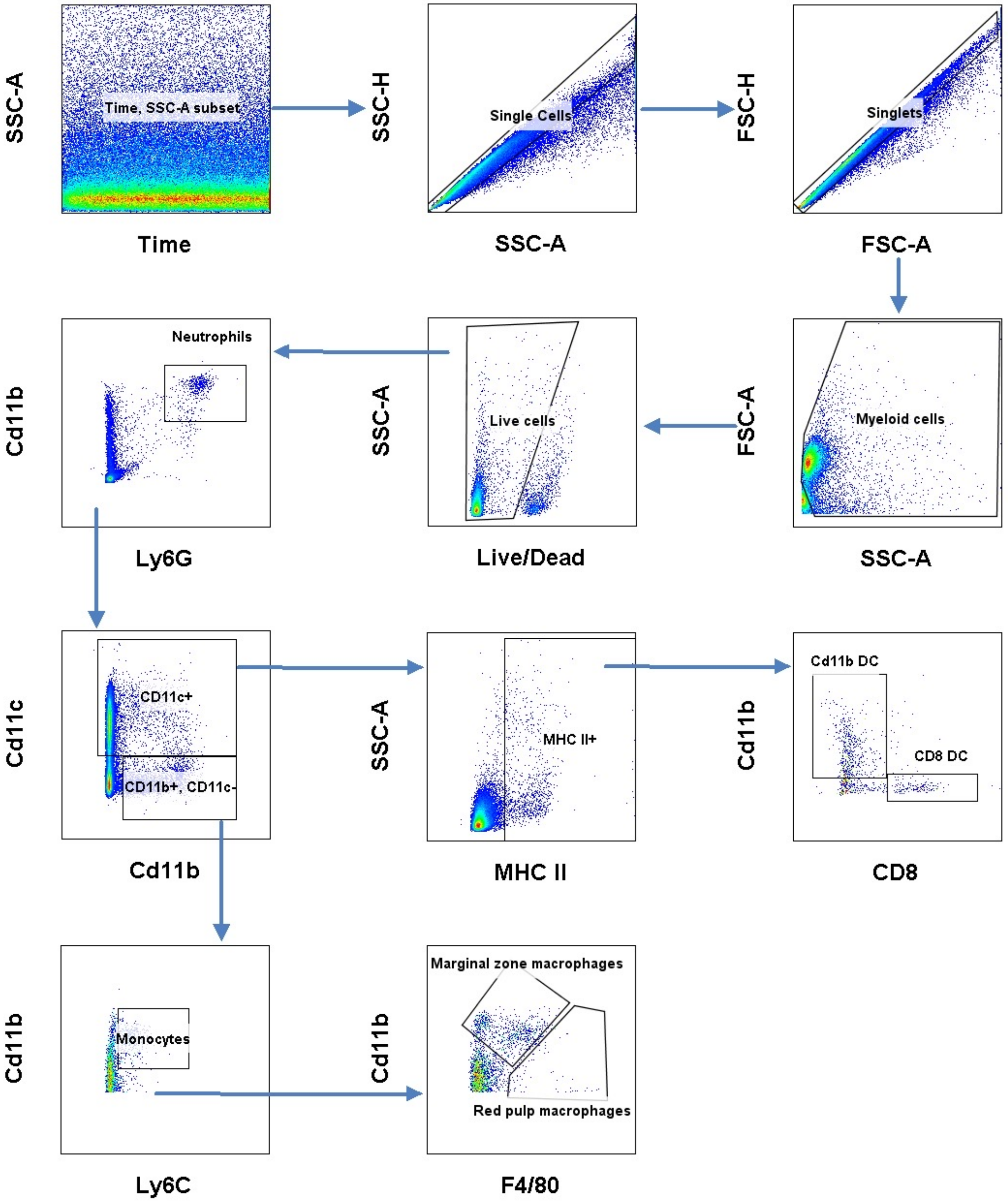
SSC-A
Time, SSC-A subset
Time
SSC-H
Single Cells
SSC-A
FSC-H
Singlets
FSC-A
Cd11b
Neutrophils
Ly6G
SSC-A
Live cells
Live/Dead
FSC-A
Myeloid cells
SSC-A
Cd11c
CD11c+
CD11b+, CD11c-
Cd11b
SSC-A
MHC II+
MHC II
Cd11b
Cd11b DC
CD8 DC
CD8
Cd11b
Monocytes
Ly6C
Cd11b
Marginal zone macrophages
Red pulp macrophages
F4/80

Figure S10

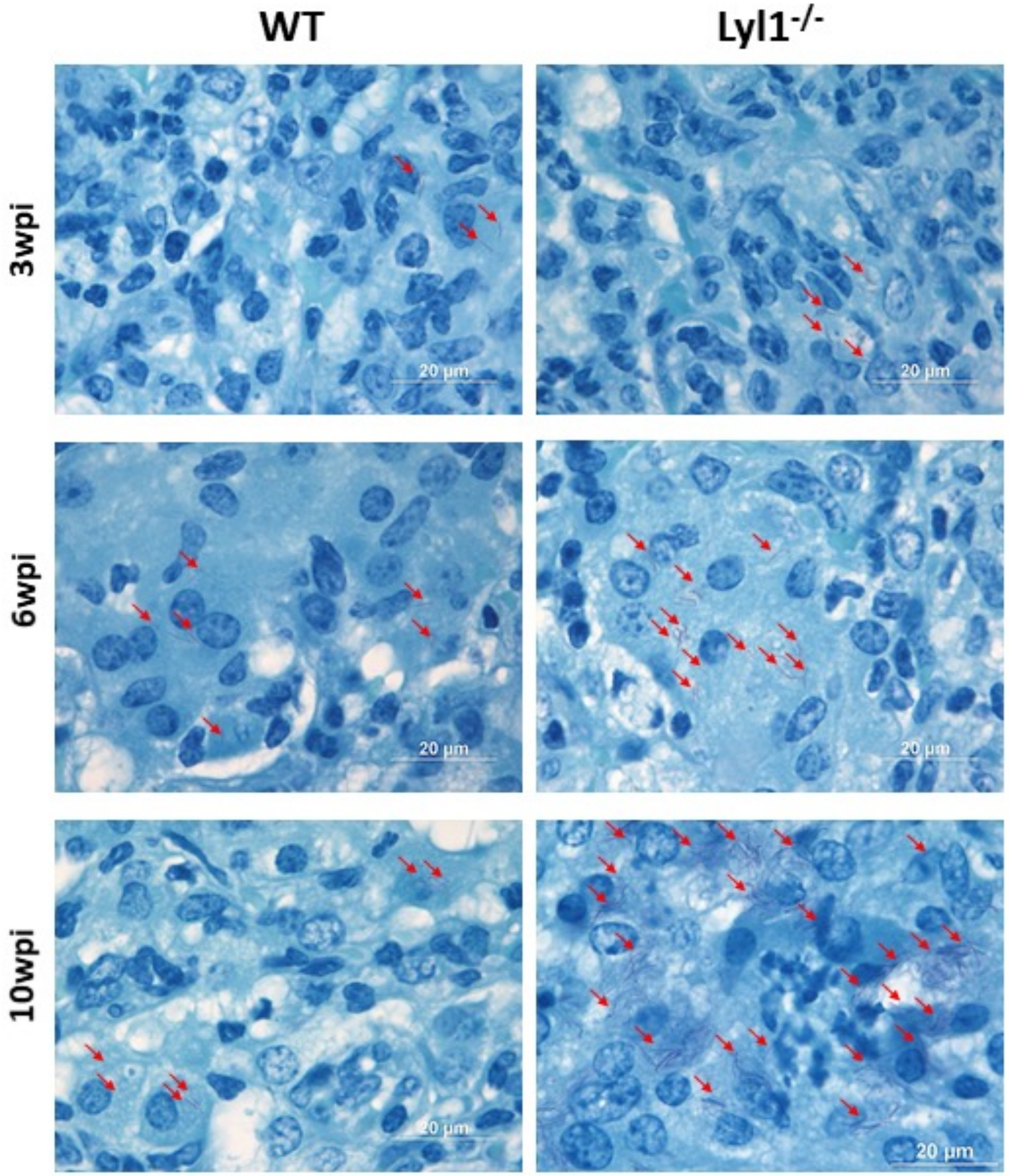

Figure S11

SUPPLEMENTARY TABLE S1

*Human Specific Gene Primers

Gene Target	Primer Sequence
Hprt – forward	5’ – GTT GGA TAT GCC CTT GAC – 3’
Hprt – reverse	5’ – AGG ACT AGA ACA CCT GCT – 3’
*HPRT – forward	5’ – AGG CGA ACC TCT CGG CTT T – 3’
*HPRT – reverse	5’ – AAG ACG TTC AGT CCT GTC CAT – 3’
Lyl1 – forward	5’ – CCC CTT CCT CAA CAG TGT CTA C – 3’
Lyl1 – reverse	5’ – TAT GGC TTG GTC TGC GCT TC – 3’
*LYL1 – forward	5’ – CAT CTT CCC TAG CAG CCG GTT G – 3’
*LYL1 – reverse	5’ – GTT GGT GAA CAC GCG CCG – 3’
Tnfa – forward	5’ – TCT CAT CAG TTC TAT GGC CC – 3’
Tnfa – reverse	5’ – GGG AGT AGA CAA GGT ACA AC – 3’
Il1a – forward	5’ – CGC TTG AGT CGG CAA AGA AAT C – 3’
Il1a – reverse	5’ – ATA CTG TCA CCC GGC TCT CC – 3’
Il1b – forward	5’ – TGC CAC CTT TTG ACA GTG ATG – 3’
Il1b – reverse	5’ – ATG TGC TGC TGC GAG ATT TG – 3’
Cxcl1 – forward	5’ – ACT GCA CCC AAA CCG AAG TC – 3’
Cxcl1 – reverse	5’ – TGG GGA CAC CTT TTA GCA TCT T – 3’
